# Association of gut microbial dysbiosis with disease severity, response to therapy and disease outcomes in Indian patients with COVID-19

**DOI:** 10.1186/s13099-023-00546-z

**Published:** 2023-05-10

**Authors:** Daizee Talukdar, Purbita Bandopadhyay, Yogiraj Ray, Shekhar Ranjan Paul, Jafar Sarif, Ranit D’Rozario, Abhishake Lahiri, Santanu Das, Debaleena Bhowmick, Shilpak Chatterjee, Bhabatosh Das, Dipyaman Ganguly

**Affiliations:** 1 Functional Genomics Laboratory, Translational Health Science and Technology Institute, Faridabad, India; 2 CSIR-Indian Institute of Chemical Biology, Kolkata, India; 3 Department of Medicine, Infectious Diseases and Beleghata General Hospital, Kolkata, India; 4 Department of Infectious Disease, SSKM Hospital, Kolkata, India; 5 Academy of Scientific and Innovative Research, Ghaziabad, India

**Keywords:** COVID-19, Gut microbiome, Dysbiosis, Cytokine, Convalescent plasma

## Abstract

**Background:**

Severe coronavirus disease 2019 (COVID-19) is associated with systemic hyper-inflammation. An adaptive interaction between gut microbiota and host immune systems is important for intestinal homeostasis and systemic immune regulation. The association of gut microbial composition and functions with COVID-19 disease severity is sparse, especially in India. We analysed faecal microbial diversity and abundances in a cohort of Indian COVID-19 patients to identify key signatures in the gut microbial ecology in patients with severe COVID-19 disease as well as in response to different therapies. The composition of the gut microbiome was characterized using 16Sr RNA gene sequences of genomic DNA extracted from faecal samples of 52 COVID-19 patients. Metabolic pathways across the groups were predicted using PICRUSt2. All statistical analyses were done using Vegan in the R environment. Plasma cytokine abundance at recruitment was measured in a multiplex assay.

**Results:**

The gut microbiome composition of mild and severe patients was found to be significantly different. Immunomodulatory commensals, viz. Lachnospiraceae family members and *Bifidobacteria* producing butyrate and short-chain fatty acids (SCFAs), were under represented in patients with severe COVID-19, with an increased abundance of opportunistic pathogens like *Eggerthella*. The higher abundance of *Lachnoclostridium* in severe disease was reduced in response to convalescent plasma therapy. Specific microbial genera showed distinctive trends in enriched metabolic pathways, strong correlations with blood plasma cytokine levels, and associative link to disease outcomes.

**Conclusion:**

Our study indicates that, along with SARS-CoV-2, a dysbiotic gut microbial community may also play an important role in COVID-19 severity through modulation of host immune responses.

**Supplementary Information:**

The online version contains supplementary material available at 10.1186/s13099-023-00546-z.

## Introduction

The novel coronavirus SARS-CoV-2 infection that led to the coronavirus disease of 2019 or COVID-19, led to a pandemic and a devastating public health crisis [1, 2]. With time, emerging variants of the virus have resulted in successive waves of community transmission worldwide [3, 4], causing close to 630 million infections that have led to approximately 6.5 million deaths till date. The unfavourable outcomes encountered in a fraction of patients mostly follow an acute respiratory distress syndrome (ARDS), which in turn follows systemic hyper-inflammation with a cytokine storm [2, 5]. Despite a lot of studies on systemic hyper-inflammation and its link to severe respiratory disease in some patients, the heterogenous immunopathology of severe COVID-19 and the indeterminacy of susceptibility to severe disease remain enigmatic [6–9]. Systemic immunosuppression using corticosteroids has been proven to be the most successful therapy [10, 11]. Anti-cytokine therapy targeting interleukin-6 is also effective in some, but not in all severe COVID-19 patients [12, 13]. Antiviral therapies using small molecules as well as combinations of antibodies have also shown limited efficacy [14–16]. Thus, meticulous charting of the systemic hyper-inflammation and the probable host-intrinsic factors playing a differential role in driving susceptibility to severity are of major importance. Moreover, a lot of variation in disease susceptibility as well as disease outcomes in different parts of the world in different cohorts of patients perhaps also points to demographic and environmental factors that shape individual host responses [17].

A crucial dimension of human heterogeneity in terms of disease risk and resilience is attributed to the commensal microbiota in different body sites [18]. The microbial ecologies of the human body have been explored in great detail in myriad noncommunicable and communicable diseases [19, 20]. With relevance to communicable infections, involvement of the gut microbiome in susceptibility to infections has been documented in bacterial infections like Salmonellosis, parasitic infections like malaria, as well as viral infections like influenza and respiratory syncytial virus [21–24] In this context, the gut microbial influence on systemic immune mechanisms has been explored in detail in different contexts [25]. Moreover, significant interest is also driven by the putative microbiome-gut-lung axis [26]. Efforts have already been put in to explore the associative link between gut microbial dysbiosis and risk-resilience balance in the context of COVID-19 [27–29].

In the present study, we analysed faecal microbiota richness, diversity, and abundance in a cohort of Indian COVID-19 patients and identified gut microbial signatures in patients with severe COVID-19 disease and their correlation with circulating cytokine abundance, as well as gut microbial changes in response to convalescent plasma therapy (CPT) and linked to disease outcomes.

## Results

### Gut microbiota dysbiosis and disease severity in patients with COVID-19

Demographic parameters, co-morbid clinical conditions, and a few baseline clinical parameters are provided in supplementary Table 1. Given the observations that SARS-CoV-2 infection may affectthe taxonomic and functional attributes of the gut microbiome, we first investigated the effect of different disease severity conditions on the faecal microbiomeof patients at the baseline level.Analysis of the data obtained from sequencing of the 16 S rRNA genes generated a total of 24, 590, 191 reads from 84 samples, with a minimum of 119, 168 and a maximum 750, 883 reads after quality filtering. The average number of reads generated was found to be 292, 740 (supplementary Table 2).

**Fig. 1:**
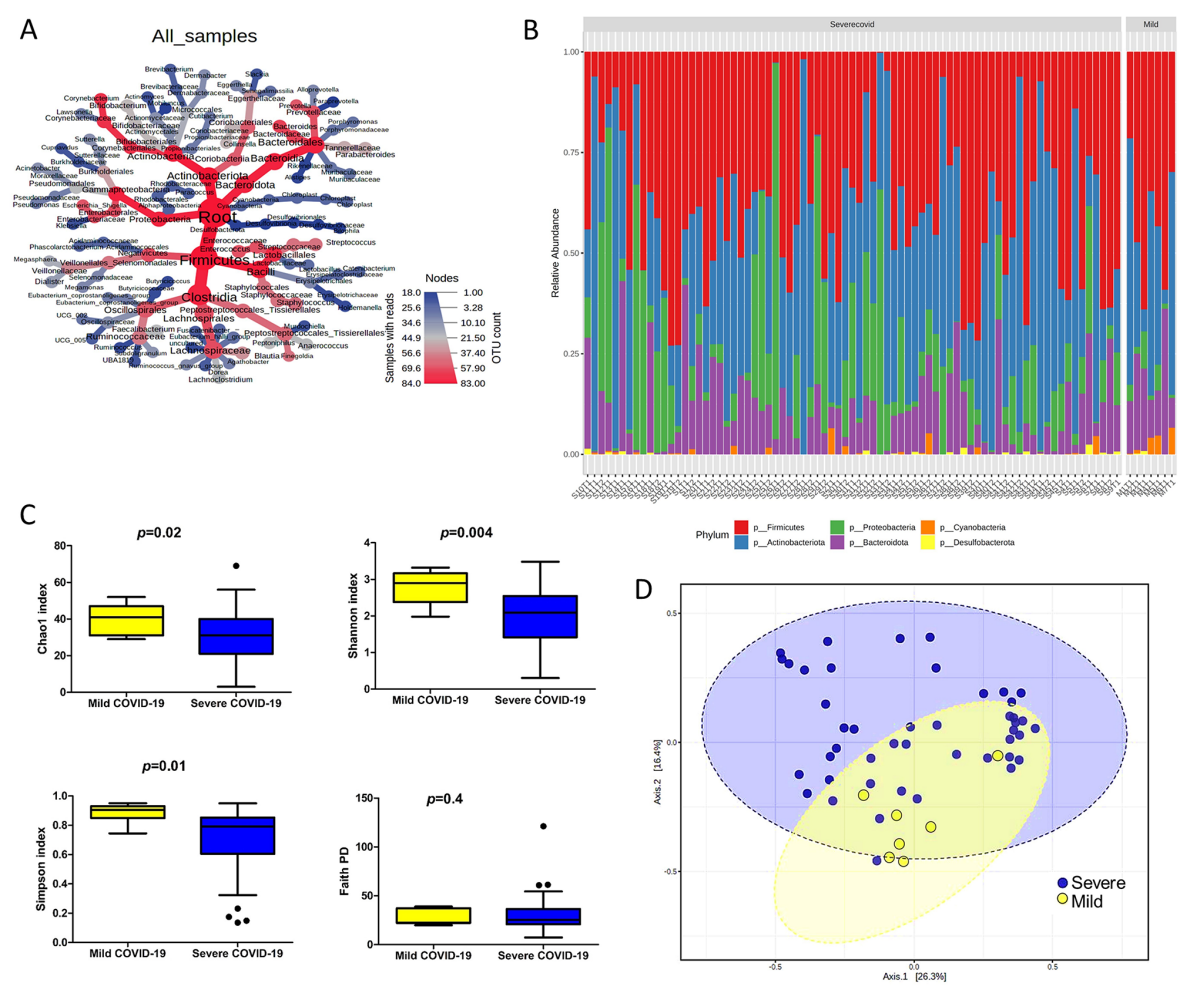
Taxonomic composition and microbial diversity in Indian patients with COVID-19 (**A**) Heat tree representing the organization of taxa from phylum through genus level. On the lower right-hand side is the colour scale. Each node represents a particular taxon used to classify the OTU whereas the edges represents where it fits in the taxonomy hierarchy. The nodes diameter is proportional to the number of OTUs and the width of the edge is equal to the number of reads. (**B**) Phylum abundance bar plot showing the relative abundance of six different phylum (Firmicutes, Proteobacteria, Bacteroides, Actinobacteria, Cyanobacteria and Desulfobacterota) in severe and Mild subjects. (**C**) Alpha diversity was found to be significant for Chao index (p-value = 0.02), Shannon index (p-value = 0.004) and Simpson index (p-value = 0.01) whearas Faith PD (p-value > 0.05) was non-significant at the baseline level. The box indicates the interquartile range (IQR). The median value is represented as a line within the box and whiskers extend to the extreme value that is within 1.5*IQR. (**D**) Beta diversity at baseline level:Principal coordinate analysis plot (PcoA) of the gut microbiome of severe and mild patients at the baseline level. The blue dots represent subjects with severe disease and yellow dots represent mild subjects. Both the groups represented on the ordination plot with a variance of 26.3% (Axis1) and 16.4% (Axis2) respectively. The statistical significance of variance between mild and severe patients was calculated using Permutational multivariate analysis of variance (PERMANOVA) (R2 = 0.0652, P-value = 0.002)

Since some features with very small counts in very few samples are likely due to sequencing errors or low-level contaminations, hence we have set the filtering criteria at a prevalence of 10%. The sequencing depth revealed a total of 20 phyla, 34 classes, 89 orders, 172 families, and 431 genera among the 84 samples. (Fig. 1A). The microbial compositionof each patient is represented in supplementary Table 3. At the phylum level it was observed that in case of severe COVID-19 patients, relative abundance of Firmicutes was the highest (39.36%) followed by Actinobacteria (33.89%), Proteobacteria (15.15%), Bacteroides (11.08%), Cyanobacteria (0.35%) and Desulfobacterota (0.14%). The relative abundance for each of the 7 Mild patients had a higher abundance of Firmicutes (43.03%), Bacteroidota (17.59%) and Cyanobacteria (2.3%), while showing considerable lower abundance of Proteobacteria (3.38%).Considering the inconsistency in sample size (Mild = 7, Severe covid = 77) these results may not reflect the actual landscape of microbial composition in COVID-19 patients, with varying disease severity. The relative abundance of these top six phylum in each sample belonging to severe and mild COVID-19 patients (n = 84) is represented in (Fig. 1B). At the baseline (time-point 1 or T1), the alpha diversity index (, Chao1, Shannon, Simpson and Faith PD) was calculated and significance was tested among the two groups as per Shapiro-Wilk test It was found that Chao1 (p-value = 0.02), Shannon (p-value = 0.004), Simpson (p-value = 0.01) were all significantly different between severe and mild COVID-19 patients), whereas, Faith PD (p > 0.05) was found to be non-significant (Fig. 1C, supplementary Table 4). In the case of beta diversity, it was observed that the Bray Curtis distance matrices showed a high significance difference between two groups at T1. Both the groups are represented on the ordination plot with a variance of 26.3% (Axis1) and 16.4% (Axis2) respectively (Fig. 1D, supplementary Table 5). Statistical significance of variance was calculated using permutational multivariate analysis of variance (PERMANOVA), which reflected that severe and mild COVID-19 patients had variance among them (R^2^ = 0.0652, p-value = 0.002).

**Fig. 2:**
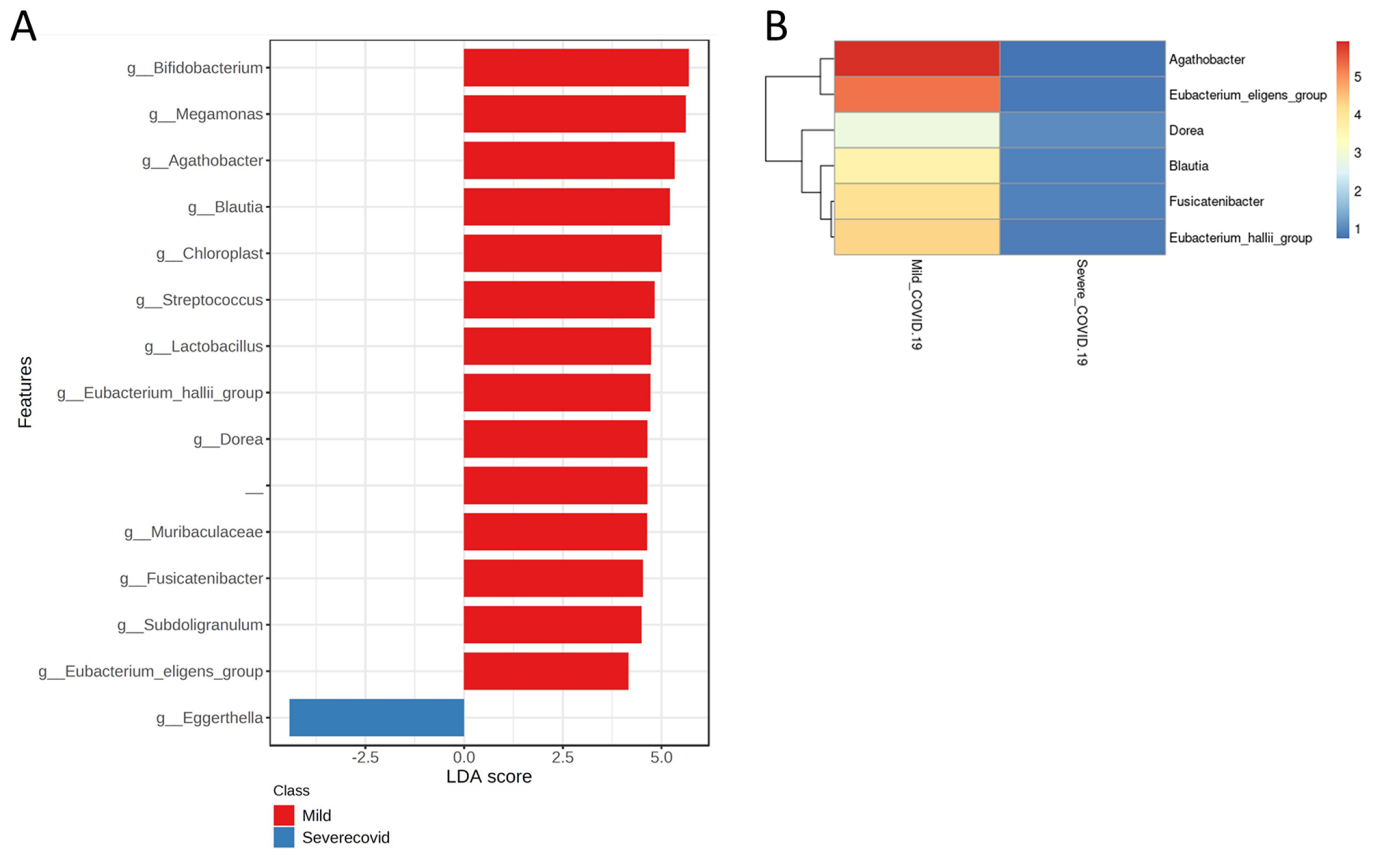
Differential abundance of gut microbiome in Mild and severe COVID-19 patients from India (**A**) LEfSe analysis identified the most differentially abundant genera (n = 15). LDA scores > 2 are shown. – in the figure is an unidentified genus from the family Lachnospiraceae. (**B**) Among the 15 genera, Lachnospiraceae family dominates the list with (n = 6) genera(p-value < 0.05, Mann Whitney U test, FDR corrected) The rows represent the mean relative abundance of each genus whereas the column stands for the mean relative abundance(%) of the two groups. Heatmap showing the bacterial composition (Lachnospiraceae family) of different COVID-19 patients (mild and severe COVID-19) at the genus level. The mean relative abundance of each bacterial genus is represented by the colour of the scale ranging from red (high mean relative abundance) to blue (low mean relative abundance) as depicted on the right side of the pheatmap. All genera belonging to Lachnospiraceae family were enriched in mild COVID-19 patients as compared to the severe population

**Fig. 3:**
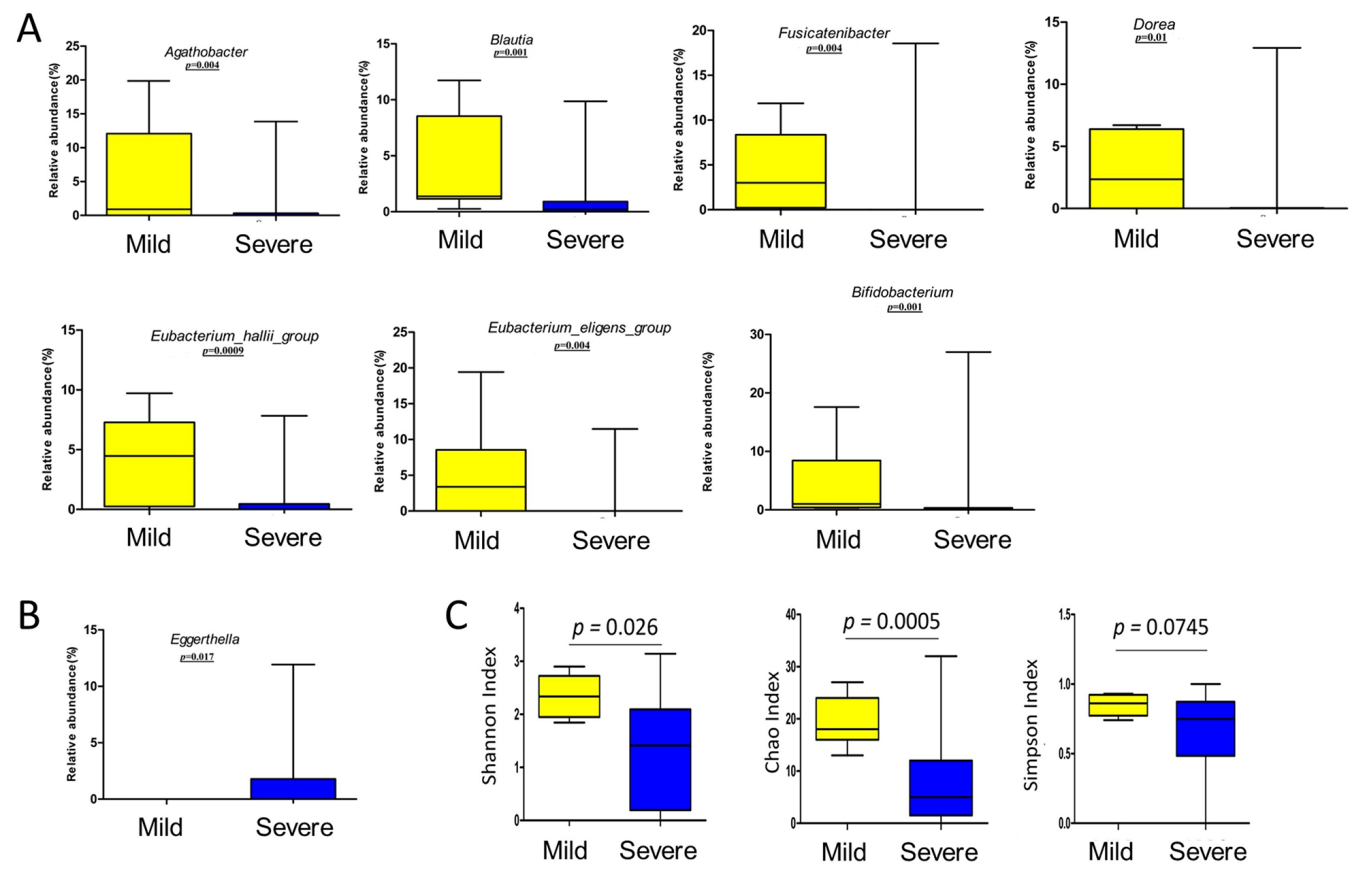
Relative abundanceof differentially abundant genera between Mild and severe COVID-19 patients from India (**A**) Box plots showing the relative abundance (%) of 6 differentially abundant generain mild and severe COVID-19 patients from the Lachnospiraceae family (p-value < 0.05, Mann Whitney U test, FDR corrected). The relative abundance (%) of genus *Bifidobacterium* was also enriched in mild patients (p-value < 0.01, Mann Whitney U test, FDR corrected). All the above-mentioned commensal gut microbes were significantly high in mild patients than in the severe disease group. (**B**) Box plots showing the relative abundance (%) of the opportunistic pathogen *Eggerthella* statistically different between the two groups (p-value < 0.05, Mann Whitney U test, FDR corrected). (**C**) Alpha diversity in the mild and severe COVID-19 patientswas computed. In Lachnospiraceae family diversity was found to be significant for Shannon index (p-value = 0.0026, Mann Whitney U test), Chao index (p-value = 0.0005 Mann Whitney U test), and non-significant for Simpson index (p-value = 0.074, Mann Whitney U test). The box indicates the interquartile range (IQR). The median value is represented as a line within the box and whiskers extend to the extreme value that is within 1.5*IQR

We extended our analysis at the genus level, to identify differentially abundant bacterial genera between the mild and severe diseasegroups. On performing FDR correction 15 genera were significantly different between the two groups. LEfSe analysis to compare the effect size between mild and severe COVID-19 groups yielded a histogram of the LDA scores which was computed for features that showed differential relative abundance between the two groups (Corrected p-value threshold = 0.1) (Fig. 2A) Six gut commensal genera belonging to Lachnospiraceae family (phylum Firmicutes) and the genus *Bifidobacterium* (phylum Actinobacteriota) were significantly enriched in patients with mild COVID-19 disease after FDR correction, viz. *Agathobacter*, *Blautia, Fusicatenibacter, Dorea, Eubacterium_halli_group* and *Eubacterium_eligens_group* (p-value < 0.05, FDR corrected). (Figures 2B and 3A, Supplementary Table 6). *Eubacterium_halli*_group, *Eubacterium_eligens*_group, *Dorea and Blautia* are well-known producers of the anti-inflammatory short-chain fatty acid (SCFA) butyrate, which is the primary metabolic fuel for colonocytes and important for maintaining colonic epithelial integrity [30]. Hence, depletion of these in severe COVID-19 patients may be relevant to disease pathogenesis. Apart from butyrate, the production of other SCFAs is mediated by bacteria such as *Bifidobacterium* species (belonging to the Phylum Actinobacteria) that produce acetate and lactate during carbohydrate fermentation.

On the other hand, opportunistic pathogens, such as *Eggerthella*, was found to be significantly enriched in severe patients (p < 0.05, FDR corrected) (Fig. 3B) in addition to *Enterococcus* and *Staphylococcus* which were more abundant in the severe group (p < 0.05, Mann Whitney U test) (Supplementary Fig. 1) but not statistically significant after FDR correction. *Staphylococcus* is a virulent pathogen that is currently the most common cause of infection in hospitalized patients [31].*Enterococci* are not detrimental on limited gut abundance ─ however transmission to other parts of the body may cause lethal infection [32]. Several studies have shown that the increased gut permeability in rheumatoid arthritis patients as well as Th17 activation in a murine colitis model are associated with an increased abundance of *Eggerthella* in the gut microbiota [33–35].

Since members of the Lachnospiraceae family were found to be the most differentially relatively abundant taxa between patients with mild and severe COVID-19, we compared the diversity in the Lachnospiraceae family between two groups of COVID-19 patients. Our analysis revealed that, Shannon (p < 0.002) and Chao (p < 0.0005) indices were statistically more prevelant in the patients with mild disease, whereas Simpson diversity did not show any statistical difference in richness and evenness between the two groups of patients (p > 0.05) (Fig. 3C, supplementary Table 7).

**Fig. 4:**
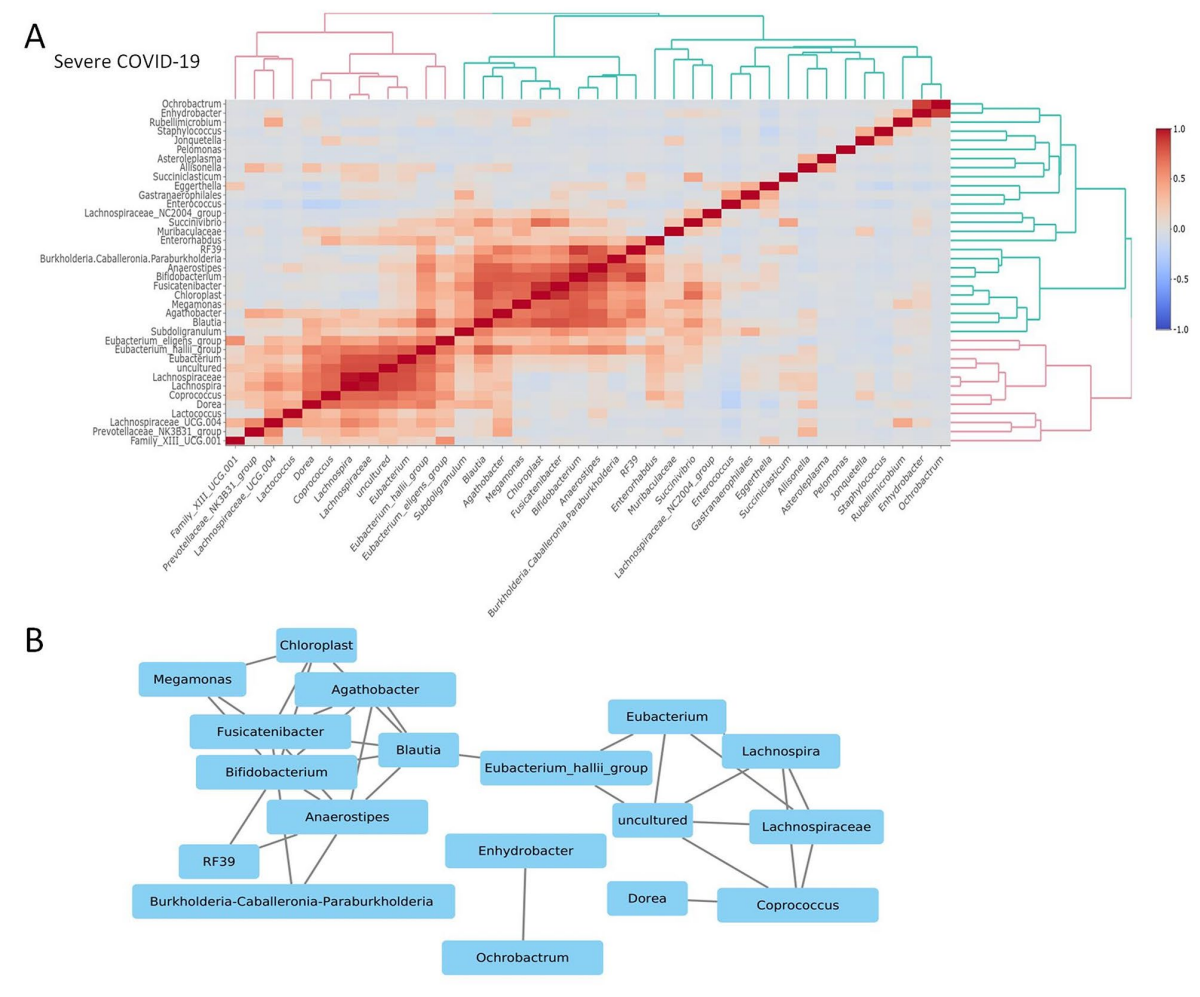
Correlation between different gut commensals and opportunistic pathogens in severe COVID-19 patients Co-occurrence among each pair of genera at T1time point in the gut microbiome was calculated using Pearson’s correlation coefficient (r). The relative abundance of the genera was used for calculation of correlation network. (**A**) Heatmap showing the correlation matrix between the different gut bacteria in patients with severe COVID-19. Red squares represent strong positive correlation blue squares represent strong negative correlation and white square represent non-significant correlations. (**B**) Co-occurrence network with threshold set to r ≥ 0.7 or r ≤ -0.7, p < 0.05 between the differentgenerain the severe COVID-19 (n = 44) group. The black lines (edges) represent strong positive interaction between the genera (signified by nodes)

Then, to gather some insight on the association between the differentially abundant genera (n = 41), we constructed the co-occurrence networks (Pearson’s correlation, with a cut-off R ≥ 0.7) and compared it between patients with mild and severe COVID-19. In patients with mild disease, the genus *Dorea* from the Lachnospiraceae family, which is a known producer of the SCFAs and butyrate [36], was found to be negatively correlated with the genera *Enhydrobacter* (Phylum Alphaproteobacteria) and *Fusicatenibacter* (Phylum Firmicutes). Besides these, the genera *Allisonella* and *Megamonas* also showed negative correlations. From these associations, we can compute that there is a strong negative correlation between butyrate and acetate-producing commensal gut bacteria and non-indigenous pathogens. Rest of the genera showed strong positive associations between them (Supplementary Fig. 2A and 2B, supplementary Table 8). In case of patients with severe COVID-19 disease, two correlative clusters were formed. The first cluster consists of 16 genera which were positively correlated with each other. The second cluster was formed by *Enhydrobacter* and *Ochrobactrum* (both belonging to Phylum Proteobacteria) showing a strong positive correlation. Both are opportunistic pathogensin the human gutcausing severe infections in immunocompetent hosts [37]. From our correlative analysis within the microbiome, we can infer that pathogenic microbes and beneficial gut bacteria have strong positive associations within themselves (Fig. 4A and B, supplementary Table 9).

### Genomic repertoire of the gut microbiota found depleted in severe COVID-19 is enriched for potentially immunomodulatory metabolic pathways

**Fig. 5:**
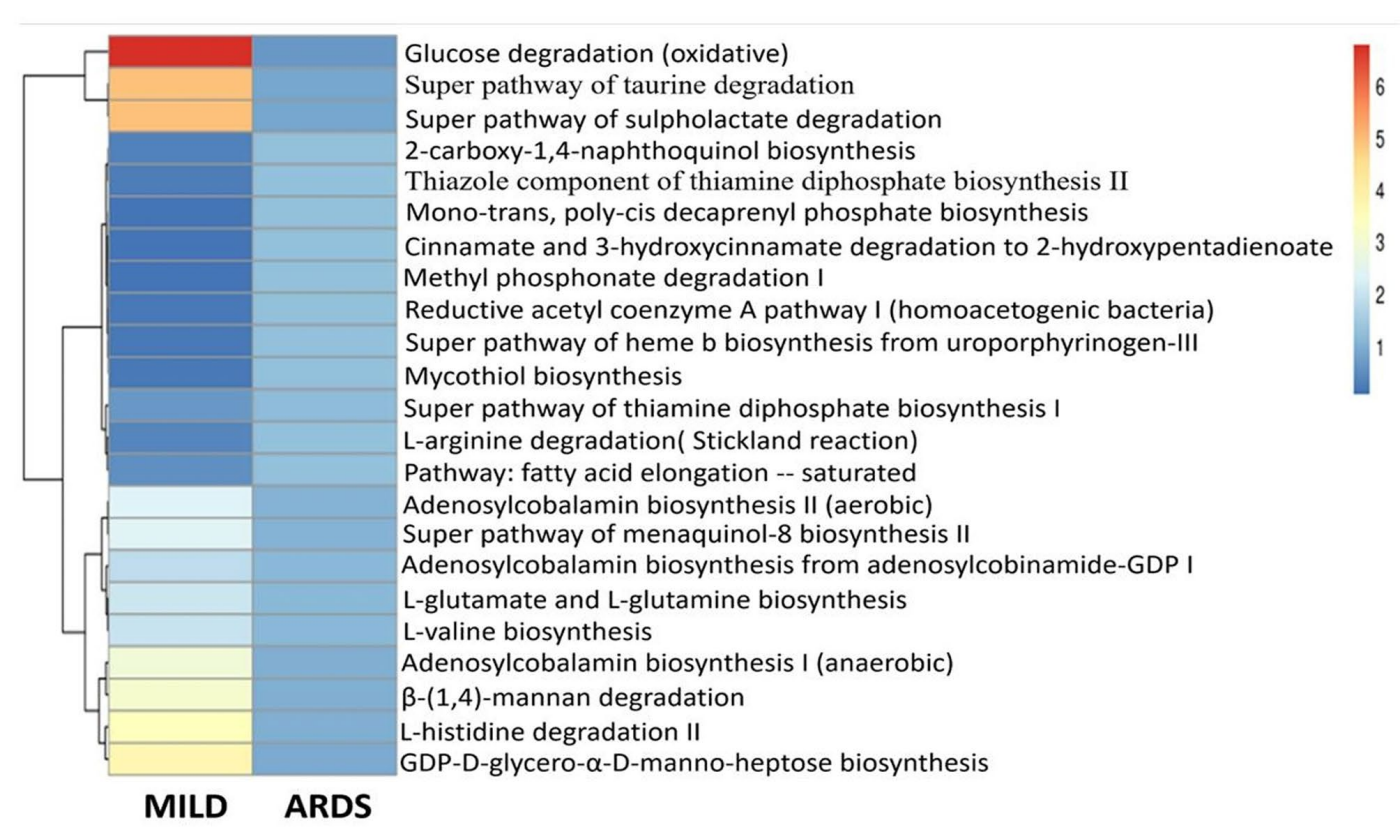
Heatmap of bacterial gene functional prediction using the latest PICRUSt2 algorithm from the faecal samples of Indian COVID-19 patients Using the amplicon sequence variants (ASV) and the biome table generated from QIIME2 database as input, PICRUSt2 predicted the KEGG-level pathways. The relative abundance was then calculated from the observed abundance of each pathway in each sample. In mild and severe COVID-19 patients a total of 23 metabolic pathways were statistically significant between the groups (p-value < 0.05, Mann Whitney U test). Among these 23 metabolic pathways, 12 were found to be highly upregulated in mild patients (p-value < 0.05, Mann Whitney U test)

Next, the metabolic capacity of the microbiome was inferred using microbial diversity obtained from the 16 S rRNA gene amplicon data by PICRUSt2 software (supplementary Table 10). The metabolic pathways of the gut microbiome were compared between different populations. The analysis revealed the differential abundance of 23 metabolic pathways inferred from the gut metagenome betweenmild and severe COVID-19 patients (p-value < 0.05, Mann Whitney U test), however, these pathways were not significantly different after computing the FDR correction using Benjamini-Hochberg method (Fig. 5, supplementary Tables 10 and supplementary Table 11). Among these pathways, twelve were found to be highly enriched for the mild disease.

Mean relative abundance of the L-histidine degradation II pathway was highly enriched in the mild COVID-19 population(mean = 3.46 in mild disease, mean = 0.98 in severe disease, p-value < 0.05, Mann Whitney U test). Catabolism of this amino acid yields glutamate and decarboxylation of histidine produces histamine. Histamine is responsible for inflammation in allergic reactions in general, but bacteria-metabolized histamine has been shown to inhibit the production of pro-inflammatory cytokines such as TNF-α in vivo and IL-1 and IL-2 in vitro [38, 39], in addition to preventing intestinal bacterial translocation.

Several amino acid metabolism pathways were also found to be highin patients with mild disease. The mean relative abundance of L-glutamate and L-glutamine biosynthesis (mean was 1.11 forsevere COVID-19, 2.06 for mild disease) and L-valine biosynthesis (mean was 1.11 forsevere COVID-19, 1.99 for mild disease) pathways were found to be under-represented in patients with severe disease (p-value < 0.05, Mann Whitney U test). In fact, various amino acids produced from the microbial protein fermentation pathways can serve as precursors for SCFA synthesis. Amino acids that are metabolized to the butyrate by anaerobic bacteria include glutamate, threonine, and lysine [40].

Cobalamin B12 biosynthesis pathways (I and II) were upregulated in the mild COVID-19 patients (mean 2.35, 2.95 in mild and 1.08, 1.02 in severe disease for I and II respectively, p-value < 0.05). Vitamin B12 serves as a co-factor for nucleotide and amino acid biosynthesis and acts as a metabolic substrate for the gut microbiome. Besides, it also provides protection against stroke in selected patients [41].

### Association of faecal microbial entities with systemic hyper-inflammation in COVID-19 patients

The systemic hyper-inflammation characterized by a differential abundance of inflammatory cytokines in response to COVID-19 infection distinguishes patients with mild symptomsfrom severe [42]. In patients with severe COVID-19, a systemic hyper-inflammatory state is encountered, associated with a cytokine storm, i.e., the large abundance of pro-inflammatory cytokines in circulation [43, 44]. This in turn may lead to septic shock and multiorgan failure [45, 46]. From our previous analyses it was apparent that gut microbiome plays an important role in disease severity and several bacteria are associated with disease severity. To explore any plausible association of the microbial dysbiosis with the nature of the cytokine storm encountered in the severe COVID-19 patients in our cohort, we ran a set of correlative analyses between the gut genera (both commensal and pathogenic)apparent from the faecal DNA amplicon sequencing data and plasma concentrations of 36 cytokines.

**Fig. 6:**
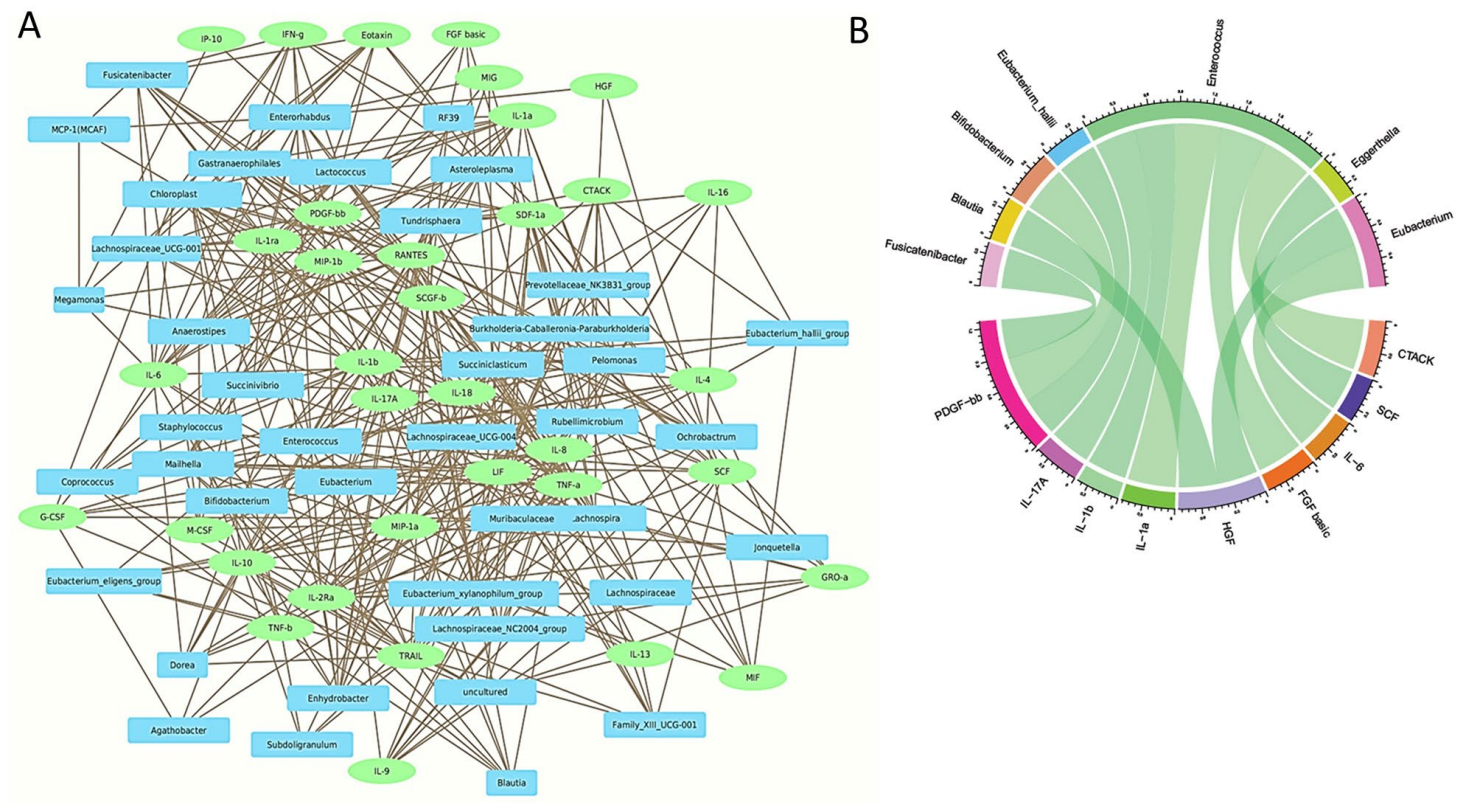
Association between gut bacteria and plasma cytokine levels in mild and severe patients (**A**) The co-occurrence among the significantly different genera and cytokines was calculated using Spearman’s rank correlation coefficient (ρ) in Mild patients.The relative abundance of the genera and log fold change (absolute values) were used for the calculation and threshold was set to r > 0.5. All calculations were done using R package, function cor(). The nodes represent genera and cytokines which are colourencoded. Green nodes represent cytokines and the blue nodes represent genera. The edges represent strong positive interaction between cytokine and genera. (**B**) Circos plot representing the co-occurrence among the significantly different genera and cytokinesin severe COVID-19 patients calculated using Spearman’s rank correlation coefficient (ρ) with the threshold set to r > 0.4. Only positive associations crossed the threshold cut-off and are represented in green where a darker shade represents stronger correlation.

To explore the plausible association of relative abundance of faecal microbial entities with absolute plasma abundance (pg/ml) of 36 cytokines we constructed a co-occurrence network (Spearman’s correlation, with a cut-off R ≥ 0.5)and compared between patients with either mild orsevere COVID-19. IL-4, IL-9, IL-10, and M-CSF were discovered to be positively associated with Lachnospira, Lachnospiraceae_UCG_004, *Eubacterium, Agathobacter, Enhydrobacter*, and *Fusicatenibacter* (Fig. 6A, supplementary Table 12). On the other hand, pro-inflammatory cytokines like IL-6, IL-8, IL-18, G-CSF, TNF-α and TNF-β were also found to be strongly correlated with several gut microbes, perhaps pointing to a feed-forward regulation. G-CSF (Granulocyte colony stimulating factor) or granulocyte production cytokine was found to be positively associated with *Agathobacter*. G-CSF, which is a growth factor that stimulates the bone marrow to produce white blood cells to reduce the risk of infection in the body, was correlated with the butyrate producing gut bacteria, which in turn is beneficial for reducing the growth of non-indigenouspathogens [47, 48].

We then checked forthe association of cytokines and gut microbes in the severe COVID-19patients. However, with correlation cut-off value of 0.5 we found that only the genus *Enterococcus* showed a positive correlation with the CC chemokine CTACK while none of the other associations crossed this cut-off R value(data not shown), supplementary Table 13). Thus, we extended our analysis by loweringthe threshold parameter to 0.4 and found significant positive associations which are represented in a circos plot in Fig. 6B.Positive correlations were observed between *Fusicatenibacter*, *Bifidobacterium* and *Eubacterium_hallii* with PDGF-bb, *Blautia* and *Eubacterium* with HGF, *Enterococcus* with CTACK, FGF basic, IL-1a IL-1b and IL-17 A, *Eggerthella* with SCF and *Eubacterium* with IL-6.

### Gut dysbiosis in response to therapy in severe COVID-19 patients

In a fraction of the severe COVID-19 patients (n = 32) faecal samples were collected 7 days post enrolment (time-point 2 or T2) and faecal DNA was analysed by 16s rRNA amplicon sequencing. We classified the patients based on whether they did or did not receive major antibiotic pharmacotherapy with or without CPT in the intervening period of one week. As reported earlier, CPT was given to the patients, randomized into the intervention arm, as two doses of 200ml of convalescent plasma on two consecutive days─ the first dose being on the day of recruitment (T1 sampling) [49]. Plasma from donors who have recovered from COVID-19 contains neutralizing antibodies against SARS-CoV-2. In addition, a prominent anti-inflammatory effect of convalescent plasma was also reported earlier, presumably due to diverse anti-inflammatory proteins circulating in the blood in the convalescence period [50].

We checked for changes in the gut microbial composition of severe COVID-19 patients before taking convalescent plasma (T1 time point, n = 15) and after administration of plasma therapy (T2 time point, n = 15). Due to small sample size FDR correction, did not yield any genus to be significantly altered between the two groups (supplementary Table 14). However, the mean relative abundance (%) of the genus Lachnoclostridium was significantly lower in the T2 time-point, i.e., post-CPT while genus Kocuria (phylum Actinobacteriota) and genus Pannonibacter (Phylum Proteobacteria) were more abundant after CPT (p-value < 0.05, Mann Whitney U test) (Supplementary Fig. 3A supplementary Table 14). *Lachnoclostridium* is known to negatively influence the circulating levels of acetate, besides being involved in the biosynthesis of detrimental lipid compounds [51]. Not much is known about the clinical significance and pathophysiology of genus *Pannonibacter* and *Kocuria*in the human gut, hence further research is needed to interpret these data.

We next analysed the effects of different therapies administered to patients with severe COVID-19 disease to understand the alteration of gut microbial composition. All the severe patients analyzed received antibiotic (Ab) and antiviral (Av) pharmacotherapy(n = 14). Hydroxychloroquine and Remdesivir, a broad-spectrum antiviral drug, were used for treating the patients. Among the other antimicrobial drugs, different antibiotics (Ab) were also variably used, viz. Doxycycline (broad spectrum tetracycline class), Clarithromycin, Azithromycin (both belong to Macrolide class), Faropenem (Beta-lactam antibiotic), Co-amoxiclav (penicillin like Ab), Levofloxacin and Norfloxacin. First, we compared the gut microbial enrichment at T1 and T2 for patients receiving only Ab and Av therapy without any CPT (Supplementary Table 15). Interestingly, potentially pathogenic genera like *Streptococcus*, *Alloprevotella* and Acinetobacter (phylum Proteobacteria), an oftenmultidrug resistant pathogen, were enriched after these therapiesat T2 timepoint. Nevertheless, a significant enrichment of the potentially metabolic health-promoting genus *Subdoligranulum* was also documented [52] (Supplementary Fig. 3B). However, observations between the two groups reflect significant mean differential relative abundance (p-value < 0.05, Mann Whitney U test), which was not evident on applying FDR correction due to the small sample size (Supplemental Table 15).

As we also had this sub-group ofpatients who also received CPT in addition to the Ab and Av therapy (all therapy, n = 13), weexplored the differential gut microbial enrichment at T2 between these two groups. As mentioned previously the sample size did not favour significant differences on FDR correction (Supplementary Table 16). However, on analysing the mean differential relative abundance between the two groups four genera belonging to the phyla Bacteroidota, Proteobacteria and Firmicutes were found to be differentially abundant at T2 between the patients receiving only antibiotic/antiviral therapy and those also receiving CPT in addition (p- value < 0.05, Mann Whitney U test). The relative abundance (%) of the genus *Acinetobacter * (phylum Proteobacteria), an often multidrug-resistant pathogen, was found to be high at T2 in patients who did not receive CPT (p-value < 0.05), whereas the relative abundance (%) of the genera * Prevotella * (phylum Bacteroidota), *Acidaminococcus* and Negativicoccus (both from Phylum Firmicutes) wereenrichedat T2 in patients also receiving CPT (p-value < 0.05, Mann Whitney U test) (Supplementary Fig. 3C, supplementary Table 16). Whether CPT had any role in the significant decline in the relative abundance (%) of *Acinetobacter*in the gut warrants further studies in larger cohorts of patients.

We next analysed the gut dysbiosis at T2among the patients taking single (n = 7), double (n = 10) or multiple combinations of antibiotics (n = 10). Altogether, we found five significantly different genera between these three groups. The relative abundance % of the genus *Holdemanella* from the phylum Firmicutes decreased as themore antibioticswere combined (p-value < 0.05, Kruskal Wallis H test). *Holdemanella* (formerly *Eubacteriumbiformis*) produces SCFAs and long chain fatty acid (LCFA) 3-hydroxyoctadecaenoic, and has been earlier reported to induce anti-cancer and anti-inflammatory effects [53]. The relative abundance of genus *Aerococcus*and *Brevundimonas* from the phylum Proteobacteria increased in patients taking more than two antibiotics (p-value < 0.05, Kruskal-Wallis H test). *Brevundimonas*was previously reported to be either hospital or community-acquired and resistant to all second and third generation antibiotics [54]. On the other hand, members of Bacteroidetes (genus *Parabacteroides*) and *Lachnoclostridium*(family Lachnospiriceae) were found to be more abundant in patients taking two antibiotics, whereas it decreased in the gut on taking more than two antibiotics (p-value < 0.05, Kruskal-Wallis H test)(Supplementary Fig. 3D, supplementary Table 17). Due to low sample size in each group, the FDR corrected values were highly conserved (> 0.05) and hence large cohort size is desirable for significant results. Thus, our analyses revealed a significant effect of antibiotic use on the differential composition of the gut microbial ecology, driving over-abundance of drug-resistant and pathogenic bacteria with multiple antibiotic usage.

### Predicted metabolic pathway enrichment in response to therapy in severe COVID-19

**Fig. 7:**
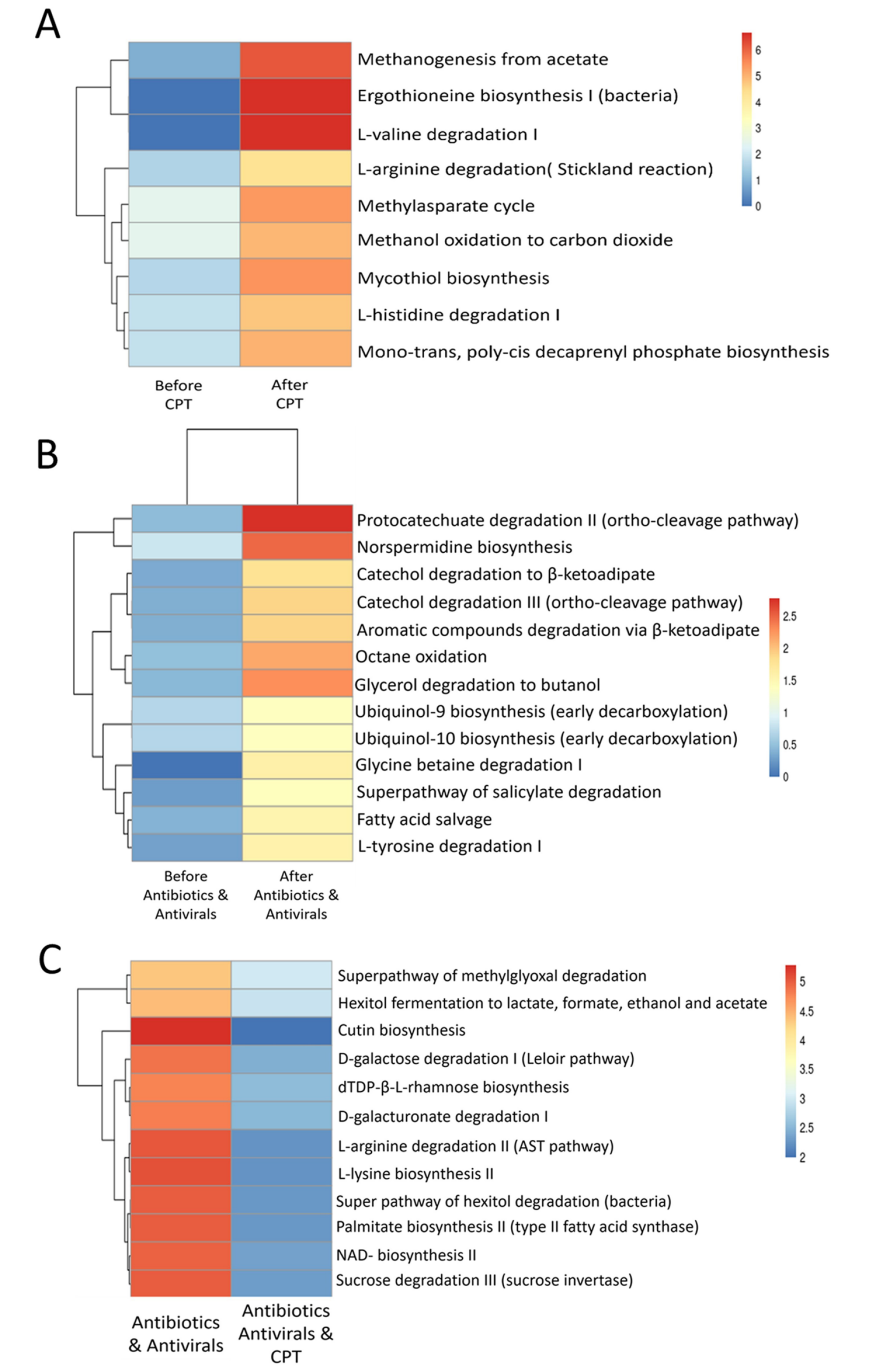
Relative abundance of metabolic pathways in response to different treatment regimes used for the treatment of COVID-19 in a cohort of Indian patients (**A**-**C**) Pheatmap of bacterial gene functional prediction using the latest PICRUSt2 algorithm from the faecal samples of patients at (**A**) T1(Before CPT) and T2 (After CPT) time point respectively. 9 metabolic pathways were differentially abundant between the two groups. (p-value < 0.05, Mann Whitney U test). (**B**) 13 metabolic pathways were differentially abundant betweensevere COVID-19patients (n = 14) at T1 time point (Before antibiotics &antivirals) and T2 time point (After antibiotics & antivirals) therapy. (p-value < 0.05, Mann Whitney U test). (**C**) 12 metabolic pathways were differentially abundant betweenpatients given antibiotic& antiviral therapy (n = 14) and the ones with antibiotic & antiviral and convalescent plasma therapy (n = 13) at T2 time point. (p-value < 0.05, Mann Whitney U test)

Next, we explored the modulation of the inferred metabolic capacity of the microbiome using microbial diversity obtained from the 16 S rRNA gene amplicon data in response to different therapies. First, we performed predicted change in thegut microbial functionality prediction in severe COVID-19 patients who received CPT (Supplementary Table 18). Our analysis revealed 9 differentially abundant pathways in the gut microbiome of patients before (pre-CPT, T1) and after (post-CPT, T2) convalescent plasma therapy (Fig. 7A, supplementary Table 19). The mean relative abundance of amino acid degradation pathways involvingL-histidine (mean = 1.82 pre-CPT at T1, mean = 4.79 post-CPT at T2) and L-valine (mean = 0.00 pre-CPT at T1, mean = 6.66 post-CPT at T2) were found to be higher in patients afterconvalescent plasma therapy (p value < 0.05, Mann Whitney U test). Various bacteria degrade proteinogenic amino acid to use them as a source of energy and nutrients [55]. Ergothioneine biosynthesis I (EGT) metabolic pathway associated with the gut microbiota was found be under-represented before plasma therapy, whereas it showed a significant upregulation on CPT(p value < 0.05, Mann-Whitney U test). The function of ergothioneine (EGT) in the microbial cells is not well understood even though it is believed that EGT may protect the cells from oxidative stress [56]. Methanol oxidation to carbon dioxide, representing detoxification, was also found to be abundant after taking the plasma therapy (p value < 0.05). Then we extended these analyses to severe COVID-19 patients who did not receive CPT and were treated only with Ab/Av therapy (Supplementary Table 20). Our analysis revealed 13 differentially abundant pathways in the gut microbiome of patients before (T1) and after (T2) 7 days of therapy with antibiotics and antivirals (Fig. 7B, supplementary Table 21).

We then compared the inferred metabolic pathway enrichment among the gut microbes between patients receiving only antibiotic and antiviral drugs (non-CPT sub-group) and patients receiving CPT in addition to along with antibiotics and antivirals (Fig. 7C, supplementary Tables 22 and 23). Carbohydrate and amino acid degradation pathways were highly representedin the gut microbiota of patients who took only antibiotic and antiviral drugs (p value < 0.05, Mann-Whitney U test). Besides these, the pathway for NAD biosynthesis (Nicotinamide adenine dinucleotide) was also found to be enriched in the non-CPT sub-group. This is the most important coenzyme in cellular redox reactions and is primarily involved in transcriptional regulation system [57]. Interestingly, mean relative abundance of the gut microbiome associated pathwaysinvolved in carbohydrate fermentation to SCFA was also higher in the non-CPT group compared with patients also receiving CPT (p value < 0.05, Maan-Whitney U test). However, on statistical computation none of these pathways had FDR corrected values < 0.05.

### Faecal microbial signature linked to disease outcomes in severe COVID-19 patients

Finally, to explore if the baseline contexture (on recruitment at T1) in the faecal microbial dysbiosis was linked with the eventual clinical outcomesin the severe COVID-19 patients, we stratified the severe COVID-19 cohort based on eventual remission (n = 31) or non-remission (death, n = 11)and ran a set of comparative analyses on their T1 genus-level gut microbiome data.

We found that the relative abundance of genus *Hungatella*, a memberof the phylum Firmicutes was significantly over-represented in patients who eventually succumbed to their severe COVID-19 disease (p value < 0.01, Mann Whitney U test) even though this was not evident after FDR correction. (Supplementary Fig. 3E , supplementary Table 24). *Hungatella hathewayi*, from the Lachnospiraceae family, is also known as *Clostridium hathewayi*. Though it is a part of the normal gut flora, it has the potential for causing bacteraemia and sepsis [58].

We computed the alpha diversity indices at the OTU level, but none of the indices showed a significant difference (Shannon, Chao1 and Simpson, p = value > 0.05) between the two groups (Supplementary Fig. 4A, supplementary Table 25). When we compared the alpha diversity indices within the Lachnospiraceae family, within the severe COVID-19 patients eventually achieving remission versus non-remission, no significant difference in Shannon, chao1 and Simpson indices (p-value > 0.05)between the two groups were observed (p-value > 0.05, Mann Whitney, U test)(supplementary Fig. 4B, supplementary Table 25). Comparative analyses of the inferred functional profiling of the gut microbial ecology between these two patient sub-groups also failed to discern a significant difference in enrichment of any metabolic pathway (p value > 0.05, Mann Whitney U test).

## Discussion

The COVID-19 epidemic has raised awareness of the importance of life and public health worldwide.This is the first study thatshows the composition of gut microbiota in an Indian cohort of COVID-19 patients and its association with disease severity and therapy outcomes.The gut dysbiosis in patients with acute respiratory distress syndrome (ARDS) is characterised by opportunistic pathogen enrichment.Several studies have shown that respiratory viral infections are associated with gut dysbiosis. For example infection by influenza and respiratory syncytial viruses has been reported to alter gut microbial composition [23]. *Staphylococcus* which is considered to be a major human opportunistic pathogen, is aprevalent cause of morbidity and mortality worldwide, known to be associated with bacteraemia, indicating susceptibility for disease severity due to potential secondary infections [59]. On the other hand, we found an increased proportion of potential pathobionts belonging to the Enterococaceaefamily(genus Enterococcus) in patients with severe COVID-19. The presence of enterococcal strains in the gut microbiome of critically ill COVID-19 patients, may act as a pool of opportunistic and antibiotic-resistant pathogens, leading to a pro-inflammatory environment in the gut [21].

In addition to this, severe COVID-19 patientsshowed a depletion in the relative abundance of several gut commensals from the dominant family of Lachnospiraceae along with the genus Bifidobacterium. These are commensal gut bacteria which produce microbial metabolites by fermenting carbohydratesand have an important role in human metabolic and immunological homeostasis [60]. The analysis of bacterial richness and alpha diversity among the groups showed that patients with mild COVID-19 disease had highermicrobial diversity than patients with ARDS and severe disease. Consistent with our results it was previously shown that there was significant depletion in the diversity of the gut microbiota in several respiratory viral infections [23]. Changes in the gut is often of fundamental importancein the pathogenesis and progression of diseases such as diabetes, obesity, autoimmune diseases and diseases related to ageing [61–63]. Patients with these diseases are established to constitute a high-risk group for developing severe COVID-19 and the associated gastrointestinal symptoms in these patients maybe indicative of COVID-19 complications [7].

Convalescent plasma therapy trials for the treatment of hospitalized patients in an attempt to ameliorate disease progression have been recognized worldwide [64]. Here we had the opportunity of analysing the changes in the gut microbiome of patients before and after convalescent plasma therapy to understand whether gut microbes could be playing any role in the disease outcome.To the best of our knowledge, these data are the first of their kind in this domain. Genus *Lachnoclostridium* was found to be depleted in the gut after convalescent plasma therapy, whereas genus *Kocuria* from Mirococacceae family, which is now considered to be a human pathogen, was found to be elevated after plasma therapy. A high abundance of the genus *Prevotella* was found in patients given all therapy. Classically, *Prevotella* strains are considered to be commensal bacterium since it is extensively present in the healthy human body, but afew strains have now been reported to give rise to opportunistic infections including pneumonia [65]. On the contrary, multidrug-resistant *Acinetobacter*, an opportunistic pathogen was relatively high in patients without convalescent plasma therapy. Furthermore, the gastrointestinal (GI) tract colonization has been linked to the development of antibiotic resistance of*A. baumannii*, presumably due to the close proximity of the organism to the enormous numbers and varieties of bacteria present [66]. The increase or decrease in the abundance of certain taxa may not be due to treatment regime alone, it might be outcome of natural course of the disease and other factors like use of dietary changes, stress, environmental expoures, etc.

The predicted metagenome analysis using PICRUSt 2 depictedmetabolic pathways that werepresent in the metagenomes of mild and severe COVID-19 patients. Gut microbiota and its metabolites have an important role in host physiology and maintenanceof immune homeostasis. Thus, from our findings, we decoded several pathways related to vitamin B12 biosynthesis, amino acid biosynthesis, amino acid degradation, and carbohydrate degradation were enriched more in mild COVID-19 patients, compared to patients with severe disease. Previous reports suggested that the functional output of the gut microbiome, mainly SCFAs and amino acids, which are considered to be important bacterial metabolites, plays a vital role in host physiology [67]. Cobalamin (Vitamin B12) has several functions including nucleotide synthesis, metabolism of long chain fatty acids and also cell metabolism, thus making it a vital factor in promoting resilience to stroke severity [41]. Decarboxylation of histidine to histamine by gut microbes hasalready been reported. This amino acid has potent immunomodulatory effects through activation of histamine receptors and the authors have stated that *L.rhamnosus* exerted anti-inflammatory effects on histamine signalling pathway activation [68].

Progression of SARS-CoV-2 infection to advanced stages is usuallyaccompanied by a vicious inflammatory response that in the future often leads to multiorgan failure. Here we have shown that the relative abundance of gut microbiota in COVID-19 patients is associated with the concentration of several blood plasma cytokines, chemokines, and inflammatory markers which thereby affect disease severity and outcomes. In a recent study, it has been reported that depletion of gut microbiota in COVID-19 cohort was linked with increasing concentrations of TNF-α, CXCL10, CCL2 and IL-10 indicating that these taxa might have a role in reducing the ARDS associated cytokine storm [69].

This study presents the first data on an Indian cohort of COVID-19 patients to examine the association of gut microbes with COVID-19 severity. One approach that can promote a healthy microbiome includes measures to increase the production of butyrate through the microbial fermentation of dietary fibres, which can mitigate the inflammatory milieu in the gut as well as systemically. Our findings highlight the fact that, along with standard therapy, modulation of the gut microbiome composition and functions with diets or other interventions may reduce the disease severity and could be a potential therapeutic opportunity for treating COVID-19 disease and its related symptoms.

Our study has a few limitations, including a relatively small sample size with mild disease, a lack of asymptomatic patients, and a non-COVID control group from the same hospital at the same time, which may have overlooked potential confounding factors. Although a large cohort size would have been desirable to firmly establish a relationship between COVID-19 severity and gut dysbiosis, the taxonomic dysbiosis identified in the present study is significant. The microbial richness and abundance differences between different taxa in the gastrointestinal tract are fairly high, so implementing the false discovery rate (FDR) for a limited sample size (< 10) with such complexity is not recommendable. In addition, the functional potency of the targeted metagenomes was predicted based on the distribution of metabolic pathways in the core genome of similar OTUs reported elsewhere but not on the basis of study samples.

## Methods

### Subject recruitment and sample collection

The patient cohort with severe COVID-19 disease (diagnosed with ARDS) was originally recruited (May, 2020 to October 2020) in a randomized control trial (RCT) for convalescent plasma therapy (CPT) in severe COVID-19 disease (CTRI/2020/05/025209), completed and reported elsewhere [49]. Patients with mild COVID-19 disease were also recruited concomitantly at the same hospital during the same time-period. The studies were done with approval of the institutional ethics committees of ID & BG Hospital (IDBGH/Ethics/2429) and CSIR-Indian Institute of Chemical Biology, Kolkata, India, in accordance with the Helsinki Declaration. Written informed consents were taken from all subjects.

### Collection of faecal samples and DNA isolation

Faecal samples were collected from 7 mild patients and 45 severe patients (Details in supplementary Table 1) immediately after enrolment (designated time-point 1 or T1) and from 32 of these severe patients (SOC n = 16, CPT n = 16), follow up faecal samples were collected 7 days post enrolment (designated time-point 2 or T2) to understand changes in the gut microbial composition between different diseased states and in response to CPT. The faecal samples were collected in 5ml 95% ethanol in PBS and stored at -20℃ until DNA isolation. For faecal DNA isolation, first the sample vials were centrifuged at 10, 000 g for 30 min and then the supernatant was discarded. The pellet was then hydrated with C1 solution of the Qiagen DNeasyPowerSoil Kit (Hilden, Germany) and the solution was then transferred to the PowerBead Tubes and the standard protocol of the kit was followed accordingly. The extracted DNA samples were stored at -20℃ until being sent for sequencing.

### Faecal DNA amplicon sequencing

Amplicon-based sequencing of the V3-V4 hyper- variable region of 16S rRNA gene with the faecal DNA samples was done by MedGenome Labs Ltd, Bangalore, India. In brief, the V3-V4 hyper-variable region of 16S rRNA gene from the isolated DNA, was amplified using universal barcoded primer pairs: V3V4F (5’CCTACGGGNGGCWGCAG’3) and V3V4R (5’GACTACHVGGGTATCTAATCC’3) [70]. Quantification of the DNA samples was done using Qubit DNA HS Assay. The DNA samples were diluted to 5ng and were amplified for 16S rRNA gene (~ 1.5 KB) using the universal 16S forward and reverse primers with a positive control and no template control in a step-up strategy by giving 35 PCR cycles. All 16S PCR products were further used to amplify V3-V4 region (~ 465 bp) using specific primers. The amplified V3-V4 PCR products were then cleaned up (1X) using AgencourtAM Pure XP beads (Beckman Coulter) so that the non-specific fragments get removed before proceeding with library preparations. The cleaned V3-V4 product was taken for library preparation using NEBNext Ultra DNA Library Prep Kit for Illumina (NEB). All the prepared libraries were checked for fragment distribution on Fragment Analyzer using HSNGS Fragment Kit (1-6000 bp). For this, the amplicon was end repaired and in a single enzymatic reaction it was mono-adenylated at 3’. The next step was ligation of the DNA fragments with NEB hairpin-loop adapters in a T4-DNA ligase-based reaction. Following ligation, USER enzyme (a combination of UDG and Endo VIII) was used for linearizing the loop containing Uracil, to make it available as a substrate for PCR based indexing in the next step. During PCR, barcodes were incorporated using unique primers for each of the samples thereby enabling multiplexing. Quantification of the prepared libraries were done using Qubit HS Assay. The qualified libraries were then sequenced on Illumina Miseq instrument to generate 0.5 M, 250BP paired end reads. All the raw sequences generated were further analyzed for taxonomic classification.

### Analyses of faecal microbiome data

The raw reads were quality controlled with Trimmomatic version 0.39 [71]. It removes adaptors and trims low quality bases from the 3’ and 5’ end of reads to generate decontaminant raw reads. It also discards trimmed reads if their length is less than 60nt. Quantitative Insights into Microbial Ecology (QIIME2, version 2018.11), a custom pipeline was used to process and analyse decontaminant raw reads [72]. Next, DADA2 package was used to demultiplex and join the paired end reads to generate long sequence [73]. This package infers exact amplicon sequence variants(ASVs) from high throughput amplicon sequencing data. Then, each sequence was assigned its taxonomy by using a pre-trained Naive Bayes classifier [74] by searching in Silva ribosomal RNA (rRNA) database release-138 [75] at 97% sequence similarity. Based on the sequence data, the taxonomy can be defined at different levels of resolution (phylum, class, order, family, genus, and species).The primary microbiome analysis was done in Marker Data Profiling (MDP) in Microbiome analyst [76, 77]. The relative abundance (%) was computed for each taxonomical hierarchy in the R version 4.1.3. The alpha diversity indices (Shannon or Shannon-Weaver, Chao1 and Simpson) were calculated using the index function present in ‘vegan’ ecological package integrated in R software [78]. The faith PD was calculated using QIIME 2 [72]. There is no general agreement as to which is the best diversity index for microbial community diversity, however Shannon and Simpson indices have been recommended to actively measure microbial diversity [79, 80]. Here in, we describe the estimates of species richness and evenness in the study of microbial communities.

### Co-occurrence network

In order to identify taxonomic groups having positive or negative influence on COVID-19 severity, pairwise correlations between the relative abundances of the commensals and pathogenic generawere computed using Pearson correlation coefficient (r) in patients with mild and severe disease separately. Similarly, co-occurrence between genera abundance and the log fold change of plasma cytokine in patients were calculated using Spearman’s rank correlation (rs) The threshold was set to r > 0.7, for the different genera in mild (n = 7) and severe disease (n = 44) conditions, and to r > 0.5, for the complete set of cytokines and differentially abundant genera in mild and severe disease separately. Cytoscape plugin Co Net was used for the construction of microbial co-occurrence network (http://apps.cytoscape.org/apps/conet) [81]. It is an available network construction method by which microbial co-occurrence are detected by putting together various association methodologies simultaneously and finally resulting in a consensus network. Wherein, the nodes represent genera and cytokines and the edges represent the, most significant association between the nodes [82].

### Predictive functional analysis

Functional predictions of the bacterial communities from the gut metagenome were computed through the Phylogenetic Investigation of Communities by Reconstruction of Unobserved States using the latest PICRUSt 2 v2.4.2 software, based on the 16 S rRNA marker using the (https://github.com/picrust/picrust2/wiki) as described by Douglas et al. in 2020 [83]. The input used was based on the unique amplicon sequence variants (ASVs) and the Biome file generated from the QIIME 2 database. Each ASVs were represented with a unique feature ID in the input files. In the first step HMMER (http://www.hmmer.org/) was used for the multiple assignment of the exact sequence variants (ESVs). The ESVs were placed in the reference tree with evolutionary placement-ng (EPA-ng) [84] and Genesis Applications for Phylogenetic Placement Analysis (gappa) omics [85] were used. A default castor R package was used for the prediction of gene families [86]. Metagenome_pipeline.py [87] was used for metagenome prediction and KEGG (Kyoto Encyclopaedia of Genes and Genomes) database was used for comparison of the output features [88]. Finally, pathway level abundances were predicted with pathway_pipeline.py function assigning EC numbers to MetaCycreactions and KO abundances in KEGG pathways [87].

### Plasma cytokine analysis

The plasma cytokine panel data, used in integrated analysis with faecal microbiome data, were generated from cryostored plasma samples, collected at recruitment, from the cohort of mild (n = 7) severe COVID-19 patients (n = 44), as previously published [49]. Multiplex cytokine analyses were done on Bioplex 200 (Biorad, Hercules, California). Out of total 48 cytokines assayed, 36 analytes, detectable in at least 70% of patients in the cohort, were finally analysed.

### Statistical analyses

The 16SrRNA gene sequences were used to characterise bacterial community composition of each sample at the genus level. The heatmap was created using Pheatmap package in R. Differences in Shannon, Chao1 index between the 2 groups were analysed by t-test (as per Shapiro-Wilk test). Whereas Simpson and FaithPD index between two groups was computed using non-parametric Wilcox test (as per Shapiro-Wilk test).For pairwise comparison between the groups Wilcox testand for more than two groups Kruskal-Wallis H test in R were used respectively.The p-values were corrected using Benjamini Hochberg method (FDR) in R environment.Intergroup differences at the genus level were analysed by linear discriminate analysis(LDA) effect size method (LEfSe) method [89].LEfSe uses the two-tailed nonparametric Kruskal-Wallis test to evaluate the significance of differences in OTUs in 2 groups.Box plots were generated in R (v4.1.3) and GraphpadPrism5. Pearsons (r) and Spearman’s rank correlation (rs) coefficient were calculated using the functions cor() or cor.test() in the R 4.1.3 environment. The statistical significance was defined as a p value < 0.05.

## Electronic supplementary material

Below is the link to the electronic supplementary material.

Supplementary Material 1

Supplementary Material 2

## Data Availability

The 16S rRNA gene sequences generated in this study has been submitted in the National Center for Biotechnology Information (NCBI) Sequence Read Archive (SRA) portaland the sequences will be available in the BioProject: PRJNA895415 (Submission ID: SUB12205249) after acceptance of the manuscript.

## References

[1] Zhu N, Zhang D, Wang W, Li X, Yang B, Song J, Zhao X, Huang B, Shi W, Lu R, et al. China Novel Coronavirus investigating and Research Team. A novel coronavirus from patients with Pneumonia in China, 2019. N Engl J Med. 2020 Feb;20(8):727–33. 10.1056/NEJMoa2001017

[2] Guan WJ, Ni ZY, Hu Y, Liang WH, Ou CQ, He JX, Liu L, Shan H, Lei CL, Hui DSC, et al. China Medical Treatment Expert Group for Covid-19. Clinical characteristics of Coronavirus Disease 2019 in China. N Engl J Med. 2020 Apr;30(18):1708–20. 10.1056/NEJMoa2002032 10.1056/NEJMoa2002032 PMC7092819 32109013

[3] Bergwerk M, Gonen T, Lustig Y, Amit S, Lipsitch M, Cohen C, Mandelboim M, Levin EG, Rubin C, Indenbaum V, et al. Covid-19 breakthrough infections in Vaccinated Health Care Workers. N Engl J Med. 2021 Oct;14(16):1474–84. 10.1056/NEJMoa2109072 10.1056/NEJMoa2109072 PMC8362591 34320281

[4] Edara VV, Pinsky BA, Suthar MS, Lai L, Davis-Gardner ME, Floyd K, Flowers MW, Wrammert J, Hussaini L, Ciric CR et al. Infection and Vaccine-Induced Neutralizing-Antibody Responses to the SARS-CoV-2 B.1.617 Variants. N Engl J Med. 2021 Aug 12;385(7):664–666. doi: 10.1056/NEJMc2107799 10.1056/NEJMc2107799 PMC8279090 34233096

[5] Mehta P, McAuley DF, Brown M, Sanchez E, Tattersall RS, Manson JJ, HLH Across Speciality Collaboration, UK. COVID-19: consider cytokine storm syndromes and immunosuppression. Lancet. 2020 Mar;28(10229):1033–4. 10.1016/S0140-6736(20)30628-0 10.1016/S0140-6736(20)30628-0 PMC7270045 32192578

[6] Bandopadhyay P, D’Rozario R, Lahiri A, Sarif J, Ray Y, Paul SR, Roy R, Maiti R, Chaudhuri K, Bagchi S et al. Nature and Dimensions of Systemic Hyperinflammation and its Attenuation by Convalescent Plasma in Severe COVID-19. J Infect Dis 2021 Aug 16;224(4):565–74. doi: 10.1093/infdis/jiab010 10.1093/infdis/jiab010 PMC7928875 34398242

[7] Li H, Liu L, Zhang D, Xu J, Dai H, Tang N, Su X, Cao B. SARS-CoV-2 and viral sepsis: observations and hypotheses. Lancet. 2020 May;9(10235):1517–20. 10.1016/S0140-6736(20)30920-X 10.1016/S0140-6736(20)30920-X PMC7164875 32311318

[8] Combes AJ, Courau T, Kuhn NF, Hu KH, Ray A, Chen WS, Chew NW, Cleary SJ, Kushnoor D, Reeder GC et al. Global absence and targeting of protective immune states in severe COVID-19. Nature. 2021 Mar;591(7848):124–30. doi: 10.1038/s41586-021-03234-7 10.1038/s41586-021-03234-7 PMC8567458 33494096

[9] Sarif J, Raychaudhuri D, D’Rozario R, Bandopadhyay P, Singh P, Mehta P, Hoque MA, Sinha BP, Kushwaha M, Sahni S et al. Plasma Gradient of Soluble Urokinase-Type Plasminogen Activator Receptor Is Linked to Pathogenic Plasma Proteome and Immune Transcriptome and Stratifies Outcomes in Severe COVID-19. Front Immunol 2021 Oct 28;12:738093. doi: 10.3389/fimmu.2021.738093 10.3389/fimmu.2021.738093 PMC8581406 34777349

[10] RECOVERY Collaborative Group, Horby P, Lim WS, Emberson JR, Mafham M, Bell JL, Linsell L, Staplin N, Brightling C, Ustianowski A, et al. Dexamethasone in hospitalized patients with Covid-19. N Engl J Med. 2021 Feb;25(8):693–704. 10.1056/NEJMoa2021436 10.1056/NEJMoa2021436 PMC7383595 32678530

[11] Dequin PF, Heming N, Meziani F, Plantefève G, Voiriot G, Badié J, François B, Aubron C, Ricard JD, Ehrmann S, et al. Effect of hydrocortisone on 21-Day mortality or respiratory support among critically ill patients with COVID-19: a Randomized Clinical Trial. JAMA. 2020 Oct;6(13):1298–306. 10.1001/jama.2020.16761 10.1001/jama.2020.16761 PMC7489432 32876689

[12] Investigators REMAP-CAP, Gordon AC, Mouncey PR, Al-Beidh F, Rowan KM, Nichol AD, Arabi YM, Annane D, Beane A, van Bentum-Puijk W, et al. Interleukin-6 receptor antagonists in critically ill patients with Covid-19. N Engl J Med. 2021 Apr;22(16):1491–502. 10.1056/NEJMoa2100433 10.1056/NEJMoa2100433 PMC7953461 33631065

[13] Salvarani C, Dolci G, Massari M, Merlo DF, Cavuto S, Savoldi L, Bruzzi P, Boni F, Braglia L, Turrà C, et al. Effect of Tocilizumab vs Standard Care on Clinical worsening in patients hospitalized with COVID-19 pneumonia: a Randomized Clinical Trial. JAMA Intern Med. 2021 Jan;181(1):24–31. 10.1001/jamainternmed.2020.6615 10.1001/jamainternmed.2020.6615 PMC7577199 33080005

[14] Cao B, Wang Y, Wen D, Liu W, Wang J, Fan G, Ruan L, Song B, Cai Y, Wei M, et al. A trial of lopinavir-ritonavir in adults hospitalized with severe Covid-19. N Engl J Med. 2020 May;7(19):1787–99. 10.1056/NEJMoa2001282 10.1056/NEJMoa2001282 PMC7121492 32187464

[15] Beigel JH, Tomashek KM, Dodd LE, Mehta AK, Zingman BS, Kalil AC, Hohmann E, Chu HY, Luetkemeyer A, Kline S, et al. Remdesivir for the treatment of Covid-19 - final report. N Engl J Med. 2020 Nov;5(19):1813–26. 10.1056/NEJMoa2007764 10.1056/NEJMoa2007764 PMC7262788 32445440

[16] Dougan M, Nirula A, Azizad M, Mocherla B, Gottlieb RL, Chen P, Hebert C, Perry R, Boscia J, Heller B, et al. Bamlanivimab plus Etesevimab in mild or moderate Covid-19. N Engl J Med. 2021 Oct;7(15):1382–92. 10.1056/NEJMoa2102685 10.1056/NEJMoa2102685 PMC8314785 34260849

[17] Gomes MGM, Ferreira MU, Corder RM, King JG, Souto-Maior C, Penha-Gonçalves C, Gonçalves G, Chikina M, Pegden W, Aguas R. Individual variation in susceptibility or exposure to SARS-CoV-2 lowers the herd immunity threshold. J Theor Biol 2022 May 7;540:111063. doi: 10.1016/j.jtbi.2022.111063 10.1016/j.jtbi.2022.111063 PMC8855661 35189135

[18] Cho I, Blaser MJ. The human microbiome: at the interface of health and disease. Nat Rev Genet. 2012 Mar 13;13(4):260 – 70. doi: 10.1038/nrg3182 10.1038/nrg3182 PMC3418802 22411464

[19] Byndloss MX, Bäumler AJ. The germ-organ theory of non-communicable diseases. Nat Rev Microbiol. 2018 Feb;16(2):103–10. 10.1038/nrmicro.2017.158 10.1038/nrmicro.2017.158 29307890

[20] Libertucci J, Young VB. The role of the microbiota in infectious diseases. Nat Microbiol 2019 Jan;4(1):35–45. doi: 10.1038/s41564-018-0278-4 10.1038/s41564-018-0278-4 30546094

[21] Velazquez EM, Nguyen H, Heasley KT, Saechao CH, Gil LM, Rogers AWL, Miller BM, Rolston MR, Lopez CA, Litvak Y, et al. Endogenous Enterobacteriaceae underlie variation in susceptibility to Salmonella infection. Nat Microbiol. 2019 Jun;4(6):1057–64. 10.1038/s41564-019-0407-8 10.1038/s41564-019-0407-8 PMC6533147 30911125

[22] Villarino NF, LeCleir GR, Denny JE, Dearth SP, Harding CL, Sloan SS, Gribble JL, Campagna SR, Wilhelm SW, Schmidt NW. Composition of the gut microbiota modulates the severity of malaria. Proc Natl Acad Sci U S A. 2016 Feb 23;113(8):2235-40. doi: 10.1073/pnas.1504887113 10.1073/pnas.1504887113 PMC4776451 26858424

[23] Yildiz S, Mazel-Sanchez B, Kandasamy M, Manicassamy B, Schmolke M. Influenza A virus infection impacts systemic microbiota dynamics and causes quantitative enteric dysbiosis. Microbiome 2018 Jan 10;6(1):9. doi: 10.1186/s40168-017-0386-z 10.1186/s40168-017-0386-z PMC5763955 29321057

[24] Harding JN, Siefker D, Vu L, You D, DeVincenzo J, Pierre JF, Cormier SA. Altered gut microbiota in infants is associated with respiratory syncytial virus disease severity. BMC Microbiol. 2020 Jun 1;20(1):140. doi: 10.1186/s12866-020-01816-5 10.1186/s12866-020-01816-5 PMC7268675 32487019

[25] Rooks MG, Garrett WS. Gut microbiota, metabolites and host immunity. Nat Rev Immunol. 2016 May 27;16(6):341 – 52. doi: 10.1038/nri.2016.42 10.1038/nri.2016.42 PMC5541232 27231050

[26] Budden KF, Gellatly SL, Wood DL, Cooper MA, Morrison M, Hugenholtz P, Hansbro PM. Emerging pathogenic links between microbiota and the gut-lung axis. Nat Rev Microbiol 2017 Jan;15(1):55–63. doi: 10.1038/nrmicro.2016.142 10.1038/nrmicro.2016.142 27694885

[27] Sarkar A, Harty S, Moeller AH, Klein SL, Erdman SE, Friston KJ, Carmody RN. The gut microbiome as a biomarker of differential susceptibility to SARS-CoV-2. Trends Mol Med. 2021 Dec;27(12):1115–34. 10.1016/j.molmed.2021.09.009 10.1016/j.molmed.2021.09.009 PMC8492747 34756546

[28] Ren Z, Wang H, Cui G, Lu H, Wang L, Luo H, Chen X, Ren H, Sun R, Liu W, et al. Alterations in the human oral and gut microbiomes and lipidomics in COVID-19. Gut. 2021 Jul;70(7):1253–65. 10.1136/gutjnl-2020-323826 10.1136/gutjnl-2020-323826 PMC8042598 33789966

[29] Nagata N, Takeuchi T, Masuoka H, Aoki R, Ishikane M, Iwamoto N, Sugiyama M, Suda W, Nakanishi Y, Terada-Hirashima J, Kimura M, Nishijima T, et al. Human gut microbiota and its metabolites Impact Immune responses in COVID-19 and its complications. Gastroenterology. 2023 Feb;164(2):272–88. 10.1053/j.gastro.2022.09.024 10.1053/j.gastro.2022.09.024 PMC9499989 36155191

[30] Parada Venegas D, De la Fuente MK, Landskron G, González MJ, Quera R, Dijkstra G, Harmsen HJM, Faber KN, Hermoso MA. Short Chain Fatty Acids (SCFAs)-Mediated Gut Epithelial and Immune Regulation and Its Relevance for Inflammatory Bowel Diseases. Front Immunol. 2019 Mar 11;10:277. doi: 10.3389/fimmu.2019.00277. Erratum in: Front Immunol. 2019 Jun 28;10:1486. 10.3389/fimmu.2019.00277 PMC6421268 30915065

[31] Kot B, Piechota M, Jakubczak A, Gryzińska M, Witeska M, Grużewska A, Baran K, Denkiewicz P. The prevalence of virulence determinants in methicillin-resistant Staphylococcus aureus isolated from different infections in hospitalized patients in Poland. Sci Rep. 2022 Mar 31;12(1):5477. doi: 10.1038/s41598-022-09517-x 10.1038/s41598-022-09517-x PMC8971418 35361858

[32] Dubin K, Pamer EG. Enterococci and Their Interactions with the Intestinal Microbiome. Microbiol Spectr. 2014 Nov;5(6):10.1128/microbiolspec.BAD-0014-2016. doi: 10.1128/microbiolspec.BAD-0014-2016. 10.1128/microbiolspec.bad-0014-2016 PMC5691600 29125098

[33] Balakrishnan B, Luckey D, Taneja V. Autoimmunity-Associated Gut Commensals Modulate Gut Permeability and Immunity in Humanized Mice. Mil Med. 2019 Mar 1;184(Suppl 1):529–536. doi: 10.1093/milmed/usy309 10.1093/milmed/usy309 30901468

[34] Kalinkovich A, Livshits G. A cross talk between dysbiosis and gut-associated immune system governs the development of inflammatory arthropathies. Semin Arthritis Rheum. 2019 Dec;49(3):474–84. 10.1016/j.semarthrit.2019.05.007 10.1016/j.semarthrit.2019.05.007 31208713

[35] Alexander M, Ang QY, Nayak RR, Bustion AE, Sandy M, Zhang B, Upadhyay V, Pollard KS, Lynch SV, Turnbaugh PJ. Human gut bacterial metabolism drives Th17 activation and colitis. Cell Host Microbe. 2022 Jan 12;30(1):17–30.e9. doi: 10.1016/j.chom.2021.11.001 10.1016/j.chom.2021.11.001 PMC8785648 34822777

[36] Vacca M, Celano G, Calabrese FM, Portincasa P, Gobbetti M, De Angelis M. The Controversial Role of Human Gut Lachnospiraceae. Microorganisms 2020 Apr 15;8(4):573. doi: 10.3390/microorganisms8040573 10.3390/microorganisms8040573 PMC7232163 32326636

[37] Ryan MP, Pembroke JT. The Genus Ochrobactrum as Major Opportunistic Pathogens. Microorganisms 2020 Nov 16;8(11):1797. doi: 10.3390/microorganisms8111797 10.3390/microorganisms8111797 PMC7696743 33207839

[38] Thomas CM, Hong T, van Pijkeren JP, Hemarajata P, Trinh DV, Hu W, Britton RA, Kalkum M, Versalovic J (2012). Histamine derived from probiotic Lactobacillus reuteri suppresses TNF via modulation of PKA and ERK signaling. PLoS ONE.

[39] Elenkov IJ, Webster E, Papanicolaou DA, Fleisher TA, Chrousos GP, Wilder RL. Histamine potently suppresses human IL-12 and stimulates IL-10 production via H2 receptors. J Immunol. 1998 Sep 1;161(5):2586-93. 9725260

[40] Barker HA (1981). Amino acid degradation by anaerobic bacteria. Annu Rev Biochem.

[41] Spence JD, Bang H, Chambless LE, Stampfer MJ. Vitamin intervention for Stroke Prevention trial: an efficacy analysis. Stroke. 2005 Nov;36(11):2404–9. 10.1161/01.STR.0000185929.38534.f3 10.1161/01.STR.0000185929.38534.f3 16239629

[42] Ling L, Chen Z, Lui G, Wong CK, Wong WT, Ng RWY, Tso EYK, Fung KSC, Chan V, Yeung ACM, Hui DSC, et al. Longitudinal Cytokine Profile in patients with mild to critical COVID-19. Front Immunol. 2021 Dec;6:12:763292. 10.3389/fimmu.2021.763292 10.3389/fimmu.2021.763292 PMC8685399 34938289

[43] Vaninov N. In the eye of the COVID-19 cytokine storm. Nat Rev Immunol. 2020 May;20(5):277. 10.1038/s41577-020-0305-6 10.1038/s41577-020-0305-6 PMC7132547 32249847

[44] Mangalmurti N, Hunter CA. Cytokine Storms: Understanding COVID-19. Immunity. 2020 Jul 14;53(1):19–25. doi: 10.1016/j.immuni.2020.06.017 10.1016/j.immuni.2020.06.017 PMC7321048 32610079

[45] Mokhtari T, Hassani F, Ghaffari N, Ebrahimi B, Yarahmadi A, Hassanzadeh G. COVID-19 and multiorgan failure: a narrative review on potential mechanisms. J Mol Histol. 2020 Dec;51(6):613–28. 10.1007/s10735-020-09915-3 10.1007/s10735-020-09915-3 PMC7533045 33011887

[46] Jose RJ, Manuel A. COVID-19 cytokine storm: the interplay between inflammation and coagulation. Lancet Respir Med. 2020 Jun;8(6):e46–7. 10.1016/S2213-2600(20)30216-2 10.1016/S2213-2600(20)30216-2 PMC7185942 32353251

[47] Alkan A, Uncu A, Taşkıran I, Tanrıverdi Ö. Double-edged sword: Granulocyte colony stimulating factors in cancer patients during the COVID-19 era. Clinics (Sao Paulo). 2020;75:e2033. doi: 10.6061/clinics/2020/e2033 10.6061/clinics/2020/e2033 PMC7330715 32638908

[48] Álvarez-Mercado AI, Navarro-Oliveros M, Robles-Sánchez C, Plaza-Díaz J, Sáez-Lara MJ, Muñoz-Quezada S, Fontana L, Abadía-Molina F. Microbial Population Changes and Their Relationship with Human Health and Disease. Microorganisms. 2019 Mar 3;7(3):68. doi: 10.3390/microorganisms7030068 10.3390/microorganisms7030068 PMC6463060 30832423

[49] Ray Y, Paul SR, Bandopadhyay P, D’Rozario R, Sarif J, Raychaudhuri D, Bhowmik D, Lahiri A, Vasudevan JS, Maurya R et al. A phase 2 single center open label randomised control trial for convalescent plasma therapy in patients with severe COVID-19. Nat Commun. 2022 Jan 19;13(1):383. doi: 10.1038/s41467-022-28064-7 10.1038/s41467-022-28064-7 PMC8770561 35046397

[50] Raychaudhuri D, Bandopadhyay P, D’Rozario R, Sarif J, Ray Y, Paul SR, Singh P, Chaudhuri K, Bhaduri R, Pandey R et al. Clinical Trial Sub-Group Analyses to Investigate Clinical and Immunological Outcomes of Convalescent Plasma Therapy in Severe COVID-19. Mayo Clin Proc Innov Qual Outcomes. 2022 Sep 14. doi: 10.1016/j.mayocpiqo.2022.09.001 10.1016/j.mayocpiqo.2022.09.001 PMC9472644 36117954

[51] Nogal A, Valdes AM, Menni C. The role of short-chain fatty acids in the interplay between gut microbiota and diet in cardio-metabolic health. Gut Microbes 2021 Jan-Dec;13(1):1–24. doi: 10.1080/19490976.2021.1897212 10.1080/19490976.2021.1897212 PMC8007165 33764858

[52] Van Hul M, Le Roy T, Prifti E, Dao MC, Paquot A, Zucker JD, Delzenne NM, Muccioli G, Clément K, Cani PD. From correlation to causality: the case of Subdoligranulum. Gut Microbes 2020 Nov 9;12(1):1–13. doi: 10.1080/19490976.2020.1849998 10.1080/19490976.2020.1849998 PMC7744154 33323004

[53] De Maesschalck C, Van Immerseel F, Eeckhaut V, De Baere S, Cnockaert M, Croubels S, Haesebrouck F, Ducatelle R, Vandamme P. Faecalicoccus acidiformans gen. nov., sp. nov., isolated from the chicken caecum, and reclassification of Streptococcus pleomorphus (Barnes 1977), Eubacterium biforme (Eggerth 1935) and Eubacterium cylindroides (Cato 1974) as Faecalicoccus pleomorphus comb. nov., Holdemanella biformis gen. nov., comb. nov. and Faecalitalea cylindroides gen. nov., comb. nov., respectively, within the family Erysipelotrichaceae. Int J Syst Evol Microbiol. 2014 Nov;64(Pt 11):3877–3884. doi: 10.1099/ijs.0.064626-0 10.1099/ijs.0.064626-0 25180093

[54] Lee MR, Huang YT, Liao CH, Chuang TY, Lin CK, Lee SW, Lai CC, Yu CJ, Hsueh PR. Bacteremia caused by Brevundimonas species at a tertiary care hospital in Taiwan, 2000–2010. Eur J Clin Microbiol Infect Dis. 2011 Oct;30(10):1185–91. 10.1007/s10096-011-1210-5 10.1007/s10096-011-1210-5 21461849

[55] Idrees M, Mohammad AR, Karodia N, Rahman A. Multimodal Role of Amino Acids in Microbial Control and Drug Development. Antibiotics (Basel). 2020 Jun 17;9(6):330. doi: 10.3390/antibiotics9060330 10.3390/antibiotics9060330 PMC7345125 32560458

[56] Cumming BM, Chinta KC, Reddy VP, Steyn AJC. Role of Ergothioneine in Microbial Physiology and Pathogenesis. Antioxid Redox Signal 2018 Feb 20;28(6):431–44. doi: 10.1089/ars.2017.7300 10.1089/ars.2017.7300 PMC5790434 28791878

[57] Lin SJ, Guarente L. Nicotinamide adenine dinucleotide, a metabolic regulator of transcription, longevity and disease. Curr Opin Cell Biol. 2003 Apr;15(2):241–6. 10.1016/s0955-0674(03)00006-1 10.1016/s0955-0674(03)00006-1 12648681

[58] Randazzo A, Kornreich A, Lissoir B. A Clostridium hathewayi isolate in blood culture of a patient with an acute appendicitis. Anaerobe. 2015 Oct;35(Pt B):44 – 7. doi: 10.1016/j.anaerobe.2015.07.003 10.1016/j.anaerobe.2015.07.003 26187681

[59] Deriu E, Boxx GM, He X, Pan C, Benavidez SD, Cen L, Rozengurt N, Shi W, Cheng G. Influenza Virus Affects Intestinal Microbiota and Secondary Salmonella Infection in the Gut through Type I Interferons. PLoS Pathog. 2016 May 5;12(5):e1005572. doi: 10.1371/journal.ppat.1005572 10.1371/journal.ppat.1005572 PMC4858270 27149619

[60] Koh A, De Vadder F, Kovatcheva-Datchary P, Bäckhed F. From Dietary Fiber to Host Physiology: Short-Chain Fatty Acids as Key Bacterial Metabolites. Cell. 2016 Jun 2;165(6):1332–1345. doi: 10.1016/j.cell.2016.05.041 10.1016/j.cell.2016.05.041 27259147

[61] Tai N, Wong FS, Wen L. The role of gut microbiota in the development of type 1, type 2 diabetes mellitus and obesity. Rev Endocr Metab Disord. 2015 Mar;16(1):55–65. 10.1007/s11154-015-9309-0 10.1007/s11154-015-9309-0 PMC4348024 25619480

[62] McLean MH, Dieguez D Jr, Miller LM, Young HA. Does the microbiota play a role in the pathogenesis of autoimmune diseases? Gut. 2015 Feb;64(2):332–41. doi: 10.1136/gutjnl-2014-308514. Epub 2014 Nov 21. 10.1136/gutjnl-2014-308514 PMC6288812 25416067

[63] Wilmanski T, Diener C, Rappaport N, Patwardhan S, Wiedrick J, Lapidus J, Earls JC, Zimmer A, Glusman G, Robinson M, Yurkovich JT, et al. Gut microbiome pattern reflects healthy ageing and predicts survival in humans. Nat Metab. 2021 Feb;3(2):274–86. 10.1038/s42255-021-00348-0 10.1038/s42255-021-00348-0 PMC8169080 33619379

[64] Avendaño-Solá C, Ramos-Martínez A, Muñez-Rubio E, Ruiz-Antorán B, Malo de Molina R, Torres F, Fernández-Cruz A, Calderón-Parra J, Payares-Herrera C, de Díaz A et al. A multicenter randomized open-label clinical trial for convalescent plasma in patients hospitalized with COVID-19 pneumonia. J Clin Invest 2021 Oct 15;131(20):e152740. doi: 10.1172/JCI152740 10.1172/JCI152740 PMC8516461 34473652

[65] Nagy E. Anaerobic infections: update on treatment considerations. Drugs. 2010 May 7;70(7):841 – 58. doi: 10.2165/11534490-000000000-00000 10.2165/11534490-000000000-00000 20426496

[66] Antunes LC, Visca P, Towner KJ. Acinetobacter baumannii: evolution of a global pathogen. Pathog Dis. 2014 Aug;71(3):292–301. 10.1111/2049-632X.12125 10.1111/2049-632X.12125 24376225

[67] Sridharan GV, Choi K, Klemashevich C, Wu C, Prabakaran D, Pan LB, Steinmeyer S, Mueller C, Yousofshahi M, Alaniz RC et al. Prediction and quantification of bioactive microbiota metabolites in the mouse gut. Nat Commun 2014 Nov 20;5:5492. doi: 10.1038/ncomms6492 10.1038/ncomms6492 25411059

[68] Frei R, Ferstl R, Konieczna P, Ziegler M, Simon T, Rugeles TM, Mailand S, Watanabe T, Lauener R, Akdis CA, et al. Histamine receptor 2 modifies dendritic cell responses to microbial ligands. J Allergy Clin Immunol. 2013 Jul;132(1):194–204. 10.1016/j.jaci.2013.01.013 10.1016/j.jaci.2013.01.013 23465664

[69] Yeoh YK, Zuo T, Lui GC, Zhang F, Liu Q, Li AY, Chung AC, Cheung CP, Tso EY, Fung KS, et al. Gut microbiota composition reflects disease severity and dysfunctional immune responses in patients with COVID-19. Gut. 2021 Apr;70(4):698–706. 10.1136/gutjnl-2020-323020 10.1136/gutjnl-2020-323020 PMC7804842 33431578

[70] Klindworth A, Pruesse E, Schweer T, Peplies J, Quast C, Horn M, Glöckner FO. Evaluation of general 16S ribosomal RNA gene PCR primers for classical and next-generation sequencing-based diversity studies. Nucleic Acids Res 2013 Jan 7;41(1):e1. doi: 10.1093/nar/gks808 10.1093/nar/gks808 PMC3592464 22933715

[71] Bolger AM, Lohse M, Usadel B. Trimmomatic: a flexible trimmer for Illumina sequence data. Bioinformatics. 2014 Aug 1;30(15):2114-20. doi: 10.1093/bioinformatics/btu170 10.1093/bioinformatics/btu170 PMC4103590 24695404

[72] Bolyen E, Rideout JR, Dillon MR, Bokulich NA, Abnet CC, Al-Ghalith GA, Alexander H, Alm EJ, Arumugam M, Asnicar F et al. Reproducible, interactive, scalable and extensible microbiome data science using QIIME 2. Nat Biotechnol. 2019 Aug;37(8):852–857. doi: 10.1038/s41587-019-0209-9. Erratum in: Nat Biotechnol. 2019 Sep;37(9):1091. 10.1038/s41587-019-0252-6 31399723

[73] Callahan BJ, McMurdie PJ, Rosen MJ, Han AW, Johnson AJ, Holmes SP. DADA2: High-resolution sample inference from Illumina amplicon data. Nat Methods 2016 Jul;13(7):581–3. doi: 10.1038/nmeth.3869 10.1038/nmeth.3869 PMC4927377 27214047

[74] Bokulich NA, Kaehler BD, Rideout JR, Dillon M, Bolyen E, Knight R, Huttley GA, Gregory Caporaso J. Optimizing taxonomic classification of marker-gene amplicon sequences with QIIME 2’s q2-feature-classifier plugin. Microbiome 2018 May 17;6(1):90. doi: 10.1186/s40168-018-0470-z 10.1186/s40168-018-0470-z PMC5956843 29773078

[75] Quast C, Pruesse E, Yilmaz P, Gerken J, Schweer T, Yarza P, Peplies J, Glöckner FO. The SILVA ribosomal RNA gene database project: improved data processing and web-based tools. Nucleic Acids Res. 2013 Jan;41(Database issue):D590–6. 10.1093/nar/gks1219 10.1093/nar/gks1219 PMC3531112 23193283

[76] Dhariwal A, Chong J, Habib S, King IL, Agellon LB, Xia J. MicrobiomeAnalyst: a web-based tool for comprehensive statistical, visual and meta-analysis of microbiome data. Nucleic Acids Res. 2017 Jul 3;45(W1):W180-W188. doi: 10.1093/nar/gkx295 10.1093/nar/gkx295 PMC5570177 28449106

[77] Chong J, Liu P, Zhou G, Xia J. Using MicrobiomeAnalyst for comprehensive statistical, functional, and meta-analysis of microbiome data. Nat Protoc. 2020 Mar;15(3):799–821. 10.1038/s41596-019-0264-1 10.1038/s41596-019-0264-1 31942082

[78] Oksanen J, Blanchet FG, Friendly M et al. Vegan: community ecology package, https://CRAN.R-project.org/package=vegan (2020, accessed 28 January 2021).

[79] Hughes JB, Hellmann JJ, Ricketts TH, Bohannan BJM. Counting the Uncountable: Statistical Approaches to Estimating Microbial Diversity. Appl Environ Microbiol. 2002 Jan;68(1):448. doi: 10.1128/AEM.68.1.448. Erratum for: Appl Environ Microbiol. 67:4399. 10.1128/AEM.67.10.4399-4406.2001 PMC93182 11571135

[80] Haegeman B, Hamelin J, Moriarty J, Neal P, Dushoff J, Weitz JS. Robust estimation of microbial diversity in theory and in practice. ISME J. 2013 Jun;7(6):1092–101. 10.1038/ismej.2013.10 10.1038/ismej.2013.10 PMC3660670 23407313

[81] Faust K, Sathirapongsasuti JF, Izard J, Segata N, Gevers D, Raes J, Huttenhower C (2012). Microbial co-occurrence relationships in the human microbiome. PLoS Comput Biol.

[82] Matchado MS, Lauber M, Reitmeier S, Kacprowski T, Baumbach J, Haller D, List M. Network analysis methods for studying microbial communities: A mini review. Comput Struct Biotechnol J 2021 May 4;19:2687–98. doi: 10.1016/j.csbj.2021.05.001 10.1016/j.csbj.2021.05.001 PMC8131268 34093985

[83] Douglas GM, Maffei VJ, Zaneveld JR, Yurgel SN, Brown JR, Taylor CM, Huttenhower C, Langille MGI. PICRUSt2 for prediction of metagenome functions. Nat Biotechnol. 2020 Jun;38(6):685–8. 10.1038/s41587-020-0548-6 10.1038/s41587-020-0548-6 PMC7365738 32483366

[84] Barbera P, Kozlov AM, Czech L, Morel B, Darriba D, Flouri T, Stamatakis A. EPA-ng: Massively Parallel Evolutionary Placement of Genetic Sequences. Syst Biol. 2019 Mar 1;68(2):365–369. doi: 10.1093/sysbio/syy054 10.1093/sysbio/syy054 PMC6368480 30165689

[85] Czech L, Stamatakis A. Scalable methods for analyzing and visualizing phylogenetic placement of metagenomic samples. PLoS One. 2019 May 28;14(5):e0217050. doi: 10.1371/journal.pone.0217050. Erratum in: PLoS One. 2019 Jul 11;14(7):e0219925. 10.1371/journal.pone.0217050 PMC6538146 31136592

[86] Louca S, Doebeli M. Efficient comparative phylogenetics on large trees. Bioinf 2018 Mar 15;34(6):1053–5. doi: 10.1093/bioinformatics/btx701 10.1093/bioinformatics/btx701 29091997

[87] Ye Y, Doak TG. A parsimony approach to biological pathway reconstruction/inference for genomes and metagenomes. PLoS Comput Biol. 2009 Aug;5(8):e1000465. 10.1371/journal.pcbi.1000465 10.1371/journal.pcbi.1000465 PMC2714467 19680427

[88] Kanehisa M, Goto S, Sato Y, Furumichi M, Tanabe M. KEGG for integration and interpretation of large-scale molecular data sets. Nucleic Acids Res. 2012 Jan;40(Database issue):D109–14. 10.1093/nar/gkr988 10.1093/nar/gkr988 PMC3245020 22080510

[89] Segata N, Izard J, Waldron L, Gevers D, Miropolsky L, Garrett WS (2011). Metagenomic biomarker discovery and explanation. Genome Biol.

