# Supplementary material for: Association of gut microbial dysbiosis with disease severity, response to therapy and disease outcomes in Indian patients with COVID-19
Source: Gut Pathog. 2023 May 10;15:22. doi: 10.1186/s13099-023-00546-z (PMC10170741; doi:10.1186/s13099-023-00546-z)
Supplement: Supplementary file 1 — Supplementary Material 1 [file 13099_2023_546_MOESM1_ESM.pdf]

Supplementary materials

For

Association of gut microbial dysbiosis with disease severity, response to therapy and disease outcomes in Indian patients with COVID-19

Talukdar D et al.

Supplementary figures 1-4 with legends

Supplementary code (Code for circus plot)

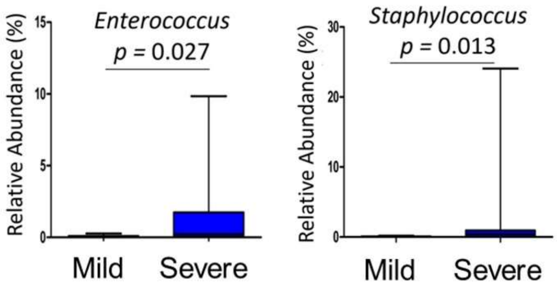

Supplementary Figure 1. Relative abundance of differentially abundant genera between Mild and severe COVID-19 patients. Box plots showing the relative abundance (%) of the opportunistic pathogens and pathobionts which were significantly higher in the severe group (Not significant in FDR correction but p-value<0.05 using Mann Whitney U test).

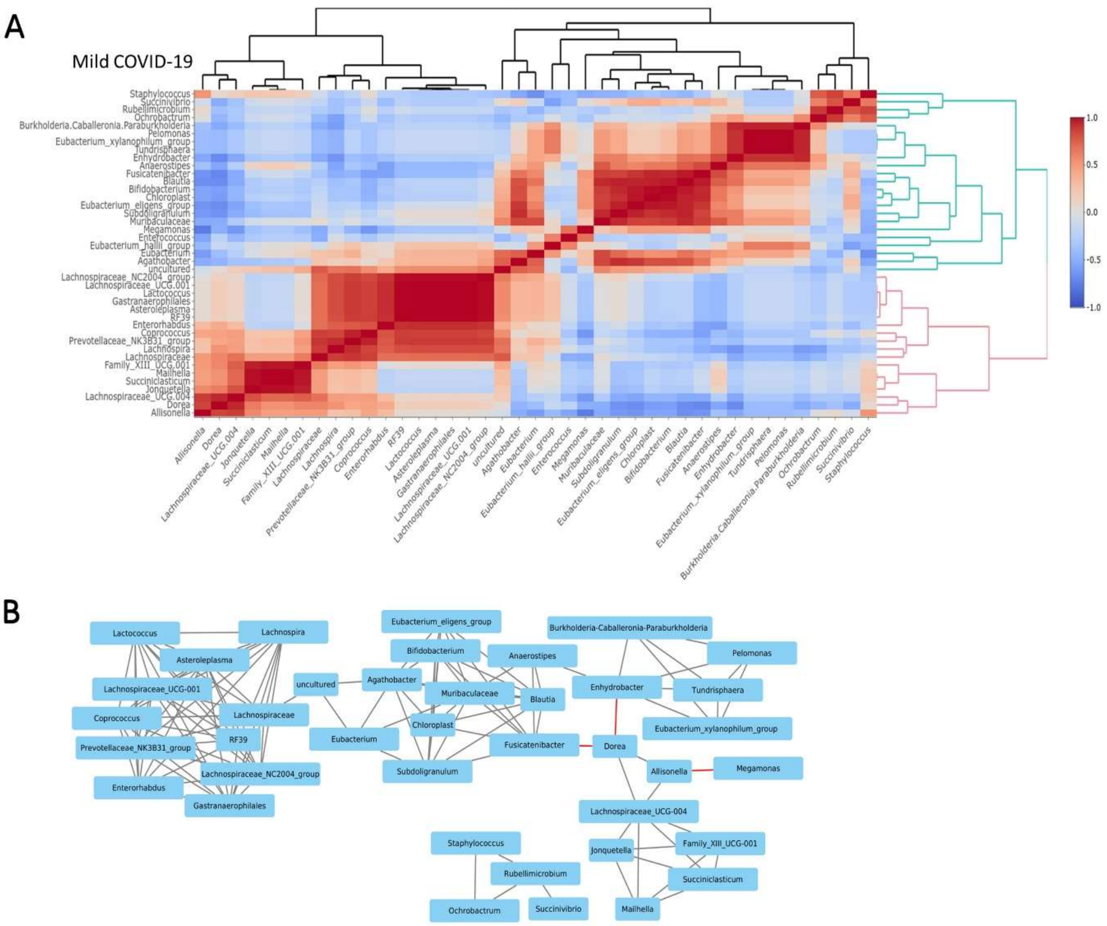

Supplementary Figure 2. Co-occurrence among each pair of genera at T1 time point in the gut microbiome was calculated using Pearson's correlation coefficient (r). The relative abundance of the genera was used for calculation of correlation network. A) Heatmap showing the correlation matrix between the significant genera ($p<0.05$) in mild group. Red squares represent strong positive correlation blue squares represent strong negative correlation and white square represent non-significant correlations. B) In the mild population, three negative associations were seen rest all are strong positive correlation Co-occurrence network was calculated with threshold set to $r \geq 0.7$ or $r \leq -0.7$, $p<0.05$ for the significantly associated genera in the mild(n=7) group. The black lines (edges) represent strong positive interaction and the red lines represent strong negative interaction between the genera (signified by nodes).

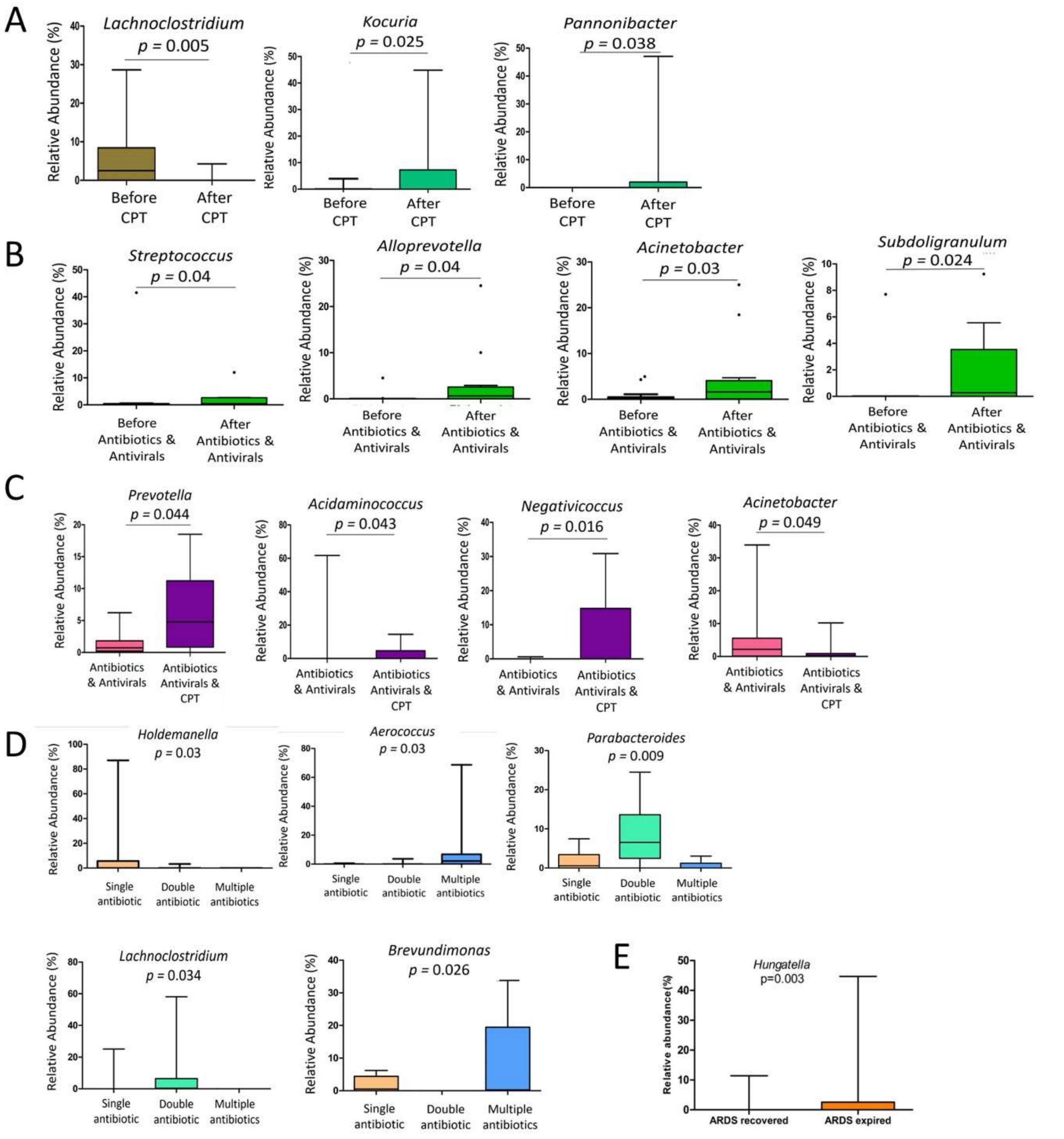

Supplementary Figure 3. Relative abundance of specific gut genera and metabolic pathways in response to different treatment regimen in COVID19 patients. The effect of convalescent plasma therapy (CPT) and antibiotics & antivirals on the change in gut microbiome is depicted in box plots showing the relative abundance (%) of the differentially abundant genera (p-value<0.05, Mann Whitney U test, not significant post FDR correction) in-: A) patients before and after convalescent plasma therapy at T1(Before CPT) and T2 (After CPT) time point respectively (n=15). A total of 3 genera were found to be statistically significant between the two groups (p-value<0.05, Mann Whitney U test). B) patients at T1 time point (Before antibiotics & antivirals) and T2 time point (After antibiotics & antivirals) therapy (n=14). 4 genera were differentially abundant between the two groups (p-value<0.05, Mann Whitney U test). C) patients given antibiotic & antiviral therapy (n=14) and patients who received CPT in addition to antibiotic & antiviral therapy (n=13) at T2 time point. Here we found 4 genera to be differentially abundant between the two groups (p-value<0.05, Mann

Whitney U test). D) patients who were given single (n=9). Double (n=10) and multiple (n=10) dose of antibiotics. 5 genera were significantly different between these three groups (p-value<0.05, Kruskal Wallis H test) at T2 time point. E) Box plot shows the relative abundance (%) of the differentially abundant genus (Hungatella from the Lachnospiraceae family) between hospitalised patients succumbing to acute respiratory distress syndrome (ARDS expired) and patients achieving remission (ARDS recovered) (p-value<0.05, Mann Whitney U test).

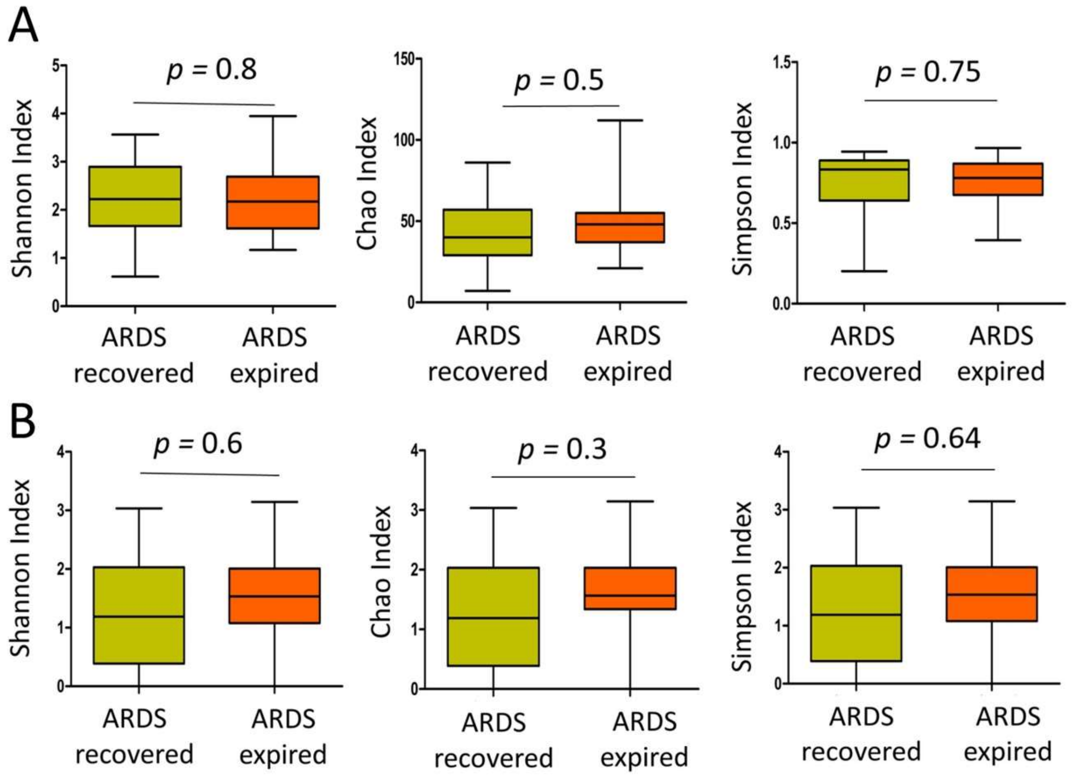

Supplementary Figure 4. A) alpha diversity indices at the OTU level (Shannon, Chao1 and Simpson, p=value>0.05) in severe COVID-19 patients achieving remission (n=31) or non-remission (Death, n=11) B) Alpha diversity indices at the OTU level within the Lachnospiraceae family (Shannon, Chao1 and Simpson, p=value>0.05) in severe COVID-19 patients achieving remission (n=31) or non-remission (Death, n=11).

Supplementary codes:

Circos plot code

```
library(circlize)

data <- read.csv("C:/Users/dclabiicb/Desktop/spearsmanR_matrix final.csv", row.names = 1)

data <- as.matrix(data)

cols <- colorRamp2(range(data), c("#000000", "#00FF83"))

chordDiagram(data, col = cols, annotationTrack = "grid", preAllocateTracks = 1)

circos.trackPlotRegion(track.index = 2, panel.fun = function(x, y) {

xlim = get.cell.meta.data("xlim")

ylim = get.cell.meta.data("ylim")

  sector.name = get.cell.meta.data("sector.index")

circos.text(mean(xlim), ylim[1] + 2, sector.name,

            facing = "clockwise", niceFacing = TRUE, adj = c(0, 0.5), cex=1.5)

circos.axis(h = "top", labels.cex = 0.5, major.tick.percentage = 0.2,

sector.index = sector.name, track.index = 2)

}, bg.border = NA)
```
